# Photochemical initiation of polariton-mediated exciton propagation

**DOI:** 10.1515/nanoph-2023-0684

**Published:** 2024-01-16

**Authors:** Ilia Sokolovskii, Gerrit Groenhof

**Affiliations:** Nanoscience Center and Department of Chemistry, University of Jyväskylä, P.O. Box 35, 40014 Jyväskylä, Finland

**Keywords:** strong light-matter coupling, exciton-polariton, quantum chemistry, quantum optics, molecular dynamics, exciton transport

## Abstract

Placing a material inside an optical cavity can enhance transport of excitation energy by hybridizing excitons with confined light modes into polaritons, which have a dispersion that provides these light–matter quasi-particles with low effective masses and very high group velocities. While in experiments, polariton propagation is typically initiated with laser pulses, tuned to be resonant either with the polaritonic branches that are delocalized over many molecules, or with an uncoupled higher-energy electronic excited state that is localized on a single molecule, practical implementations of polariton-mediated exciton transport into devices would require operation under low-intensity incoherent light conditions. Here, we propose to initiate polaritonic exciton transport with a photo-acid, which upon absorption of a photon in a spectral range not strongly reflected by the cavity mirrors, undergoes ultra-fast excited-state proton transfer into a red-shifted excited-state photo-product that can couple collectively with a large number of suitable dye molecules to the modes of the cavity. By means of atomistic molecular dynamics simulations we demonstrate that cascading energy from a photo-excited donor into the strongly coupled acceptor-cavity states via a photo-chemical reaction can indeed induce long-range polariton-mediated exciton transport.

## 1 Introduction

Organic opto-electronic materials offer many advantages over their silicon counterparts, such as lower production cost, smaller weight, higher flexibility and easier disposability, but are hampered by low exciton mobility [1]. Enhancing that mobility therefore has become a major optimization target and several solutions have been proposed, which include increasing the lifetime via triplet formation [2], [3], ordering molecules to increase exciton delocalization [[Bibr j_nanoph-2023-0684_ref_004] [Bibr j_nanoph-2023-0684_ref_005] [Bibr j_nanoph-2023-0684_ref_006], or coupling the excitons to the confined light modes of an optical resonator [[Bibr j_nanoph-2023-0684_ref_008] [Bibr j_nanoph-2023-0684_ref_009] [Bibr j_nanoph-2023-0684_ref_010] [Bibr j_nanoph-2023-0684_ref_011] [Bibr j_nanoph-2023-0684_ref_012] [Bibr j_nanoph-2023-0684_ref_013] [Bibr j_nanoph-2023-0684_ref_014] [Bibr j_nanoph-2023-0684_ref_015]. Because the latter solution does not require chemical modifications of the molecules, which may compromise other properties, utilizing strong light–matter coupling could be a promising route towards improving the performance of organic opto-electronic devices.

**Figure 1:**
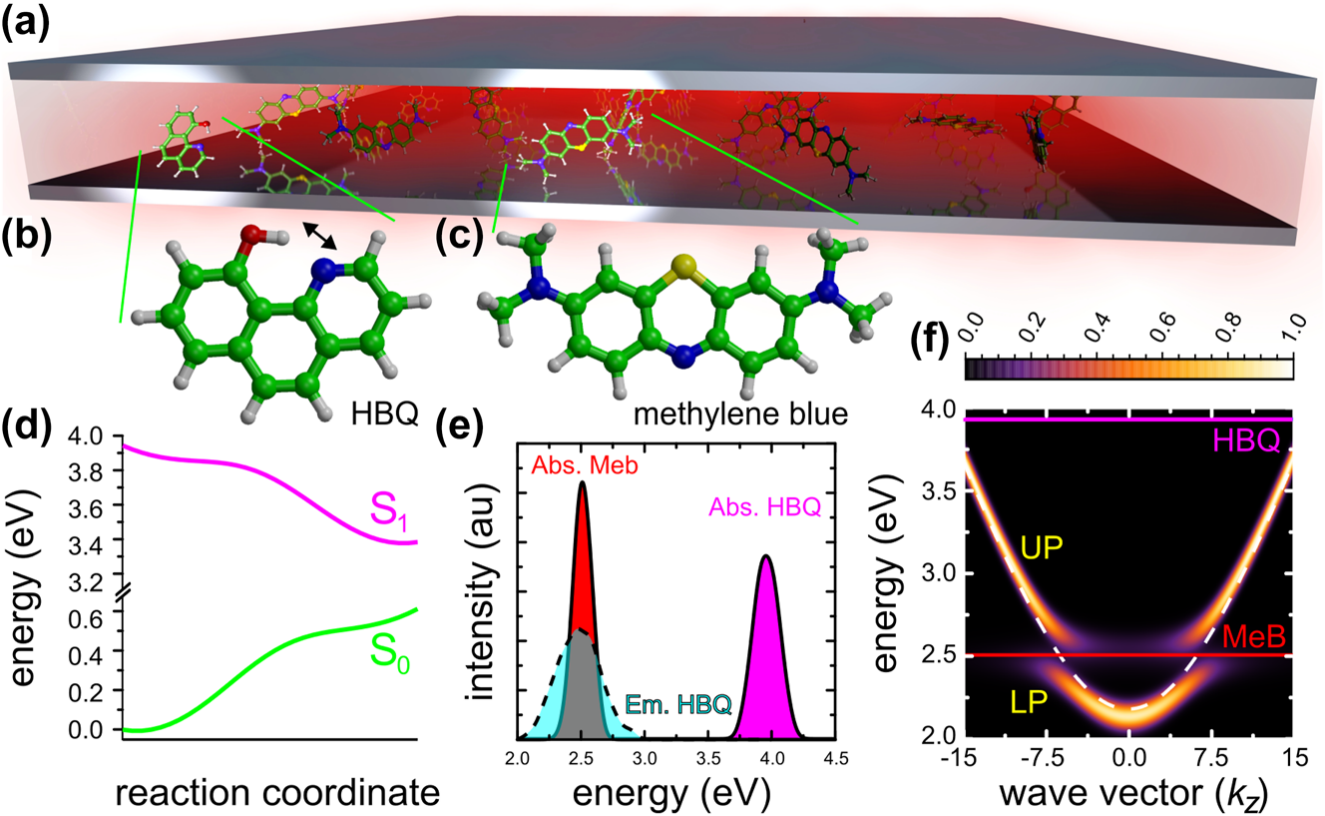
Illustration of a Fabry–Pérot microcavity (panel (a), not to scale) containing a 10-hydroxybenzo[h]quinoline donor molecule (HBQ, panel (b)) and 1023 methylene blue acceptor molecules (MeB, panel (c)). The first singlet excited states (S_1_) of the MeB molecules are coupled to the 239 modes of the cavity. Upon absorbing a photon at a frequency where the mirrors have become more transparent (∼3.96 eV at the TDA-CAMB3LYP/3-21G level of theory, Figure S4 in SM), the uncoupled HBQ undergoes ultra-fast intra-molecular proton transfer on the S_1_ excited-state potential energy surface (panel (d)) into an excited-state photo-product that is resonant with both the absorption maximum of MeB and the cavity. Panel (e) shows the normalised QM/MM absorption (magenta) and emission (cyan) spectra of HBQ and the absorption spectrum of MeB (red). The normalised angle-resolved absorption spectrum of the molecule-cavity system (panel (f)) shows the Rabi splitting of 282 meV between the lower polariton (LP) and upper polariton (UP) branches. The cavity dispersion is plotted as a white dashed line, while the excitation maxima of the MeB molecules (∼2.5 eV at the TD-B97/3-21G level of theory) and HBQ are plotted as straight red and magenta lines, respectively.

Because the confinement of light into smaller volumes by an optical resonator increases the interaction with molecular transitions [17], the enhanced exciton mobility in the strong coupling regime has been attributed to hybridization of excitons and confined light modes into polaritons [[Bibr j_nanoph-2023-0684_ref_018] [Bibr j_nanoph-2023-0684_ref_019] [Bibr j_nanoph-2023-0684_ref_020] [Bibr j_nanoph-2023-0684_ref_021] [Bibr j_nanoph-2023-0684_ref_022], which can form when the interaction strength exceeds the decay rates of both excitons and cavity modes [24], [25]. The hybrid states with contributions from cavity modes are bright and can hence be accessed optically [26], [27], [28]. Because the cavity mode energy depends on the in-plane momentum, or wave-vector, *k*_*z*_, these states have dispersion and form the upper and lower polaritonic branches, as shown in Figure 1f. These branches are separated by the Rabi splitting, which is defined as the energy gap at the wave-vector for which the energy of the exciton and cavity dispersion is resonant. Most of the hybrid states, however, have negligible contribution from the cavity modes, and are hence dark [29]. These dark states therefore also lack dispersion and form a quasi-degenerate manifold instead that is situated in between the two bright polaritonic branches.

Owing to their dispersion, the bright polaritonic states support ballistic propagation of population at their group velocity (*i.e.*, *v*_*g*_ = ∂*ω*(*k*_*z*_)/∂*k*_*z*_, with *ℏω*(*k*_*z*_) the energy of a polariton with in-plane momentum *k*_*z*_, Figure S3 in Supplementary Material, SM) [15], [18], [19], [22], [23], [30], [31]. However, while in inorganic micro-cavities, such ballistic propagation was indeed observed [32], [33], transport in organic micro-cavities is a diffusion process because of rapid dephasing in disordered organic materials [19]. Results from molecular dynamics (MD) simulations suggest that such dephasing is due to reversible exchange of population between the stationary dark states and propagating polaritonic states [13], [34], [35]. Although polariton-mediated exciton transport is not ballistic in organic systems, polaritonic diffusion can still dramatically outperform the intrinsic exciton diffusivity of the material [9], [16]. However, despite several experimental realizations [8], [9], ], and an emerging theoretical understanding of polariton propagation in organic microcavities [, [30], [31], [36], [37], strong light–matter coupling has so far not been leveraged systematically for practical applications.

**Figure 2:**
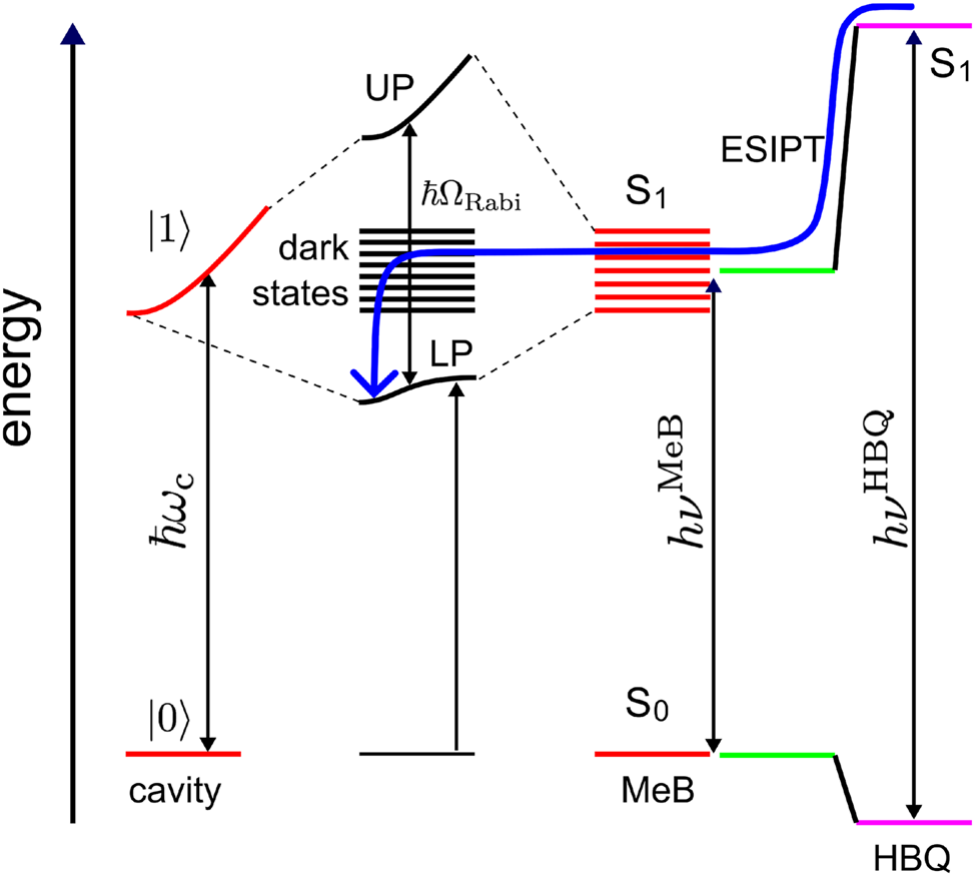
Simplified Jablonski diagram of states involved in photo-chemically induced polariton propagation. The cavity modes and polaritons are schematically shown as continuous dispersions. After photo-excitation of HBQ at *hν* ^HBQ^, excited-state intra-molecular proton transfer (ESIPT) brings the excited state population that was initially localized on HBQ, into the dark state manifold. Reversible population exchanges between the stationary dark states and the propagating lower polaritonic (LP) bright states cause the population to move away from the HBQ molecule and diffuse into the cavity [34]. The path along which the population arrives in the LP states is illustrated by a blue arrow.

One of the obstacles on the path to polaritonic devices for enhanced exciton transfer is that polariton propagation requires laser excitation of either wavepackets of polaritonic states [11], [14], or higher-energy electronic states of the molecules [, [16]. Yet, for practical applications, such as light-harvesting, it will be essential that transport can also be initiated with low-intensity incoherent light sources. To address this specific challenge for a Fabry–Pérot optical resonator, we propose to initiate polariton propagation in a strongly coupled molecule-cavity system with a suitable donor that, upon excitation at wavelengths for which the cavity mirrors are transparent [38], undergoes a rapid photo-chemical reaction into an excited-state photo-product with an emission maximum that is resonant with both the cavity and acceptor dye molecules. As illustrated in Figures 1 and 2, such system could potentially be realized if we combine 10-hydroxybenzo[h]quinoline (HBQ) that upon excitation at 375 nm or 360 nm undergoes ultra-fast excited-state intra-molecular proton transfer (ESIPT) on a femtosecond timescale into a photo-product with a broad emission centered at 620 nm [39], [40], with methylene blue (MeB) in an optical micro-cavity made of silver mirrors and resonant with the broad absorption peaks of MeB at 668 nm or 609 nm (Figure S4, in the Supplementary Material). Here, we demonstrate the feasibility of this concept by means of hybrid quantum mechanics/molecular mechanics (QM/MM) molecular dynamics simulations [41], [42].

## 2 Materials and methods

### 2.1 Multiscale Tavis–Cummings model

We performed QM/MM MD simulations [43], [44] of one HBQ molecule solvated in cyclohexane and 1023 hydrated MeB molecules strongly coupled to the confined light modes of a one dimensional Fabry–Pérot micro-cavity (Figure S1) [45]. Within the Born–Oppenheimer approximation, we separate the nuclear degrees of freedom, which we treat classically, from the electronic plus cavity degrees of freedom, which we treat quantum mechanically with the QM/MM extension of the traditional Tavis–Cummings model of quantum optics [46], [47]. In the Supplementary Material (SM), we provide a concise description of our multi-scale simulation approach, which was presented in detail in previous publications [41], [42], [48].

### 2.2 HBQ model

In the QM/MM Tavis–Cummings Hamiltonian, the electronic ground (S_0_) and excited (S_1_) states of HBQ were modeled with density functional theory (DFT) [49] and time-dependent density functional theory (TDDFT) [50] within the Tamm–Dancoff approximation (TDA) [51], respectively, using the CAM-B3LYP functional [52], [53] in combination with the 3-21G basis set [54]. The cyclohexane solvent molecules were modelled with the GROMOS 2016H66 force field [55]. At this level of theory, the vertical excitation energy of HBQ is *hν* ^HBQ^ = 3.96 eV (312 nm) with a full width at half maximum (FWHM) of 270 meV, while the energy gap to the ground state is 2.58 eV (480 nm, 500 meV FWHM) in the S_1_ minimum (Figure 1e). Despite the overestimation of the S_1_–S_0_ energy gap with respect to experiment (3.3 eV for absorption and 2.0 eV for emission), our model provides potential energy surfaces (Figure 1d) that are in qualitative agreement with the more accurate description at the TPSSh/cc-pVDZ level of theory for this system (Figure S5 in SM) [56], [57].

### 2.3 MeB model

The S_0_ and S_1_ electronic states of MeB were modelled with DFT and TDDFT based on the Casida equations [58], respectively, using the B97 functional [59] and the 3-21G basis set. The water molecules were described with the TIP3P model [60]. Although at this level of theory, the vertical excitation energy of MeB is *hν* ^MeB^ = 2.5 eV (210 meV FWHM), and thus significantly overestimated with respect to experiment, there is a fortuitous overlap with the emission of HBQ that we exploit in this work (Figure 1e). Thus, while MeB may not be the optimal choice for a practical realization, this dye should be suitable for demonstrating the feasibility of inducing polariton-mediated exciton transport with a photo-chemical reaction in our simulations. Further details of the HBQ and MEB simulation setups are provided in the SM.

### 2.4 HBQ/MeB cavity model

From a QM/MM trajectory of HBQ in the S_0_ state, single HBQ snapshots were selected and combined with 1023 frames from a QM/MM trajectory of MeB in S_0_. These 1024 molecules, including their solvent environments, were placed at equal inter-molecular separations on the *z*-axis of a 1D [45], 50 μm long, symmetric optical Fabry–Pérot micro-cavity (Figure S1), with HBQ at the center of the cavity (*i.e.*, *z*_HBQ_ = 25 μm). In our setup, *z* indicates the in-plane direction (*i.e.*, parallel to the mirrors). With a distance of *L*_*x*_ = 284 nm between the mirrors (cavity width), where *x* indicates the out-of-plane direction (*i.e.*, perpendicular to the mirrors), the fundamental mode of the cavity has an energy of *ℏω*_0_ = 2.18 eV at normal incidence (*i.e.*, *k*_*z*_ = 0) and hence its dispersion is red-detuned by 320 meV with respect to the Methylene Blue absorption maximum at 2.5 eV (vertical red line in Figure 1f). The dispersion of the cavity was modelled with 239 discrete modes (*i.e.*, *k*_*z*,*p*_ = 2*πp*/*L*_*z*_ with −119 ≤ *p* ≤ 119 and *L*_*z*_ = 50 μm). To maximize the collective light–matter coupling strength, the transition dipole moments of all molecules were aligned to the vacuum field, which is parallel to the *y*-axis of the cavity, at the start of the simulation. With a vacuum field strength of **E**_*y*_ = 0.00004 (0.21 MVcm^−1^) the Rabi splitting, defined as the energy gap between the UP and LP at the wave vector where the molecular excitation energy matches the cavity mode energy, is 282 meV. Simulations were performed for a lossy cavity with a decay rate of ℏ*γ*_cav_ = 0.04 eV or *γ*_cav_ = 66.7 ps^−1^. At such rate, the lifetime, *τ*_cav_, of the lossy cavity is comparable to the 2–14 fs lifetimes of metallic Fabry–Pérot cavities used in experiments on strong coupling with organic molecules [9], [61], [62]. With a lifetime of 15 fs and a resonance at 2.18 eV, the quality-factor, defined as *Q* = *ω*_cav_ *τ*_cav_, would be 50 for our cavity. In addition, we also performed simulations in a better cavity with a decay rate of *γ*_cav_ = 10 ps^−1^, as well as an ideal cavity with an infinite lifetime (*i.e.*, *γ*_cav_ = 0 ps^−1^).

Because the molecules do not interact directly, but rather via the cavity modes, there are no issues in using different QM/MM descriptions for HBQ and MeB. Although the solvents, as well as the force fields and QM methods, were chosen because of convenience, we emphasize that for the purpose of this work it is not essential to have the most accurate description of the bare excitation energies of the molecules, but rather to have a realistic model of the molecular degrees of freedom, including the solvent environment. We speculate that for practical realizations HBQ could be embedded via small micro-droplets of a suitable solvent within the polymer matrix containing MeB, or *vice versa*.

### 2.5 Molecular dynamics simulations

Ehrenfest MD trajectories were computed by numerically integrating Newton’s equations of motion using a leap-frog algorithm with a 0.5 fs time step [63]. At each time step, the multi-mode Tavis–Cummings Hamiltonian (Equation (1) in SM) is constructed in the basis of product states between the *N* molecular excitations, obtained from QM/MM calculations [64], and the *n*_mode_ cavity mode excitations:

(1) $$\left|\phi_{j}\rangle ={\hat{\sigma }}_{j}^{+}|{\text{S}}_{0}^{1}{\text{S}}_{0^{\;..}}^{2}{\text{S}}_{0}^{N-1}{\text{S}}_{0}^{N}\rangle ⊗|00..0\right\rangle$$

for 1 ≤ *j* ≤ *N*, and

(2) $$\left|\phi_{j>N}\rangle ={\hat{a}}_{j-N}^{†}|{\text{S}}_{0}^{1}{\text{S}}_{0^{\;..}}^{2}{\text{S}}_{0}^{N-1}{\text{S}}_{0}^{N}\rangle ⊗|00..0\right\rangle$$

for *N* < *j* ≤ *N* + *n*_mode_. In these expressions, $\left|{\text{S}}_{0}^{i}\right\rangle$ indicates that molecule *i* is in the electronic ground state, while |00..0⟩ indicates that the Fock states for all *n*_mode_ cavity modes are empty. The operators ${\hat{\sigma }}_{j}^{+}$ and ${\hat{a}}_{p}^{†}$ excite molecule *j* and cavity mode *p*, respectively. Owing to the orthogonality of the electronic states and of the cavity modes, this basis is strictly diabatic within the single excitation subspace [48].

The total wave function, |Ψ(*t*)⟩, was coherently propagated along with the classical degrees of freedom of the molecules as a time-dependent superposition of these diabatic product states (Equations (1) and (2)):

(3) $$|\Psi \left(t\right)〉=\underset{j}{\sum }c_{j}\left(t\right)|\phi_{j}〉$$

with *c*_*j*_(*t*) the time-dependent expansion coefficients of the time-independent basis states |*ϕ*_*j*_⟩. To account for the finite lifetime of the cavity modes, the wave function was propagated along the classical trajectory under the influence of an effective non-Hermitian Hamiltonian (SM), in which losses were added to the cavity mode energies (*i.e.*, *ℏω*(*k*_*z*_) − *i*/2ℏ*γ*_cav_, with *ℏω*(*k*_*z*_) the dispersion of the empty cavity, shown as a dashed white line in Figure 1f) [65], [66], [67], [68].

To obtain also the adiabatic polaritonic eigenstates [46], [47], defined as

(4) $$\left|\psi^{m}\rangle =\left(\sum_{j}^{N}\beta_{j}^{m}{\hat{\sigma }}_{j}^{+}+\sum_{p}^{n_{\text{mode}}}\alpha_{p}^{m}{\hat{a}}_{p}^{†}\right)|{\text{S}}_{0}^{1}{\text{S}}_{0^{\;..}}^{2}{\text{S}}_{0}^{N-1}{\text{S}}_{0}^{N}\rangle |0\right\rangle$$

and required for analysis and the determination of the initial conditions for the simulations, we diagonalized the QM/MM Tavis–Cummings Hamiltonian (Equation (1) in SM). The $\beta_{j}^{m}$ and $\alpha_{p}^{m}$ expansion coefficients reflect the contribution of the molecular excitons $\left(\left|{\text{S}}_{1}^{j}\right\rangle \right)$ and of the cavity mode excitations (|1_*p*_⟩) to the adiabatic state |*ψ* ^*m*^⟩. All simulations were initiated in the highest-energy adiabatic state, *m* = 1263, for which $|\beta_{\text{HBQ}}^{1263}{|}^{2}\approx 1$ .

The simulations were performed with GROMACS version 4.5.3 [69], in which the multi-mode Tavis–Cummings QM/MM model was implemented [42], in combination with Gaussian16 [70]. The GROMACS source code is available for download from https://github.com/upper-polariton/GMXTC. The results presented in the main manuscript are averages over four trajectories, started with different initial conditions.

## 3 Results and discussion

**Figure 3:**
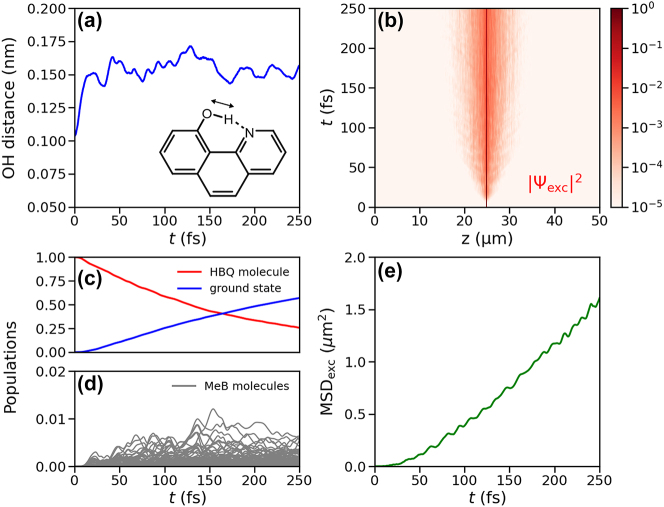
Panel (a) shows the distance between the oxygen and proton, which we use as the reaction coordinate for the excited-state proton transfer reaction (inset and see also Figure 1b) as a function of time after instantaneous excitation into the highest energy eigenstate of the molecule-cavity system, which is dominated by the *S*_1_ state of HBQ (*i.e.*, |*β*_HBQ_|^2^ ≈ 0.99, panel (c)). Panel (b) depicts the probability density of the excitonic part of the wave function |Ψ_exc_|^2^ as a function of the *z*-coordinate (horizontal axis) and time (vertical axis). Panels (c) and (d) show the contribution of the HBQ molecule (red) and the Methylene Blue molecules (grey) to the total wave function, as well as the population of the ground state (blue). Panel (e) shows the mean squared displacement (*i.e.*, MSD_exc_ = ⟨*z*(*t*) − *z*(0)⟩^2^) of the excitonic wavepacket.

In Figure 3, we plot the progress of the excited-state intra-molecular proton transfer (ESIPT) reaction in HBQ, defined as the distance between the hydroxyl oxygen and the proton (a), the excitonic part of the total wavepacket |Ψ_exc_(*z*, *t*)|^2^ (b), the contributions of the molecular excitations to the total wave function, |Ψ(*z*, *t*)|^2^ (c, d), and the mean squared displacement of the excitonic wavepacket (MSD_exc_, e). After photo-excitation into the highest-energy eigenstate of the molecule-cavity system (*i.e.*, |*ψ* ^1263^⟩, which is dominated by the S_1_ electronic state of HBQ (*i.e.*, $|\beta_{\text{HBQ}}^{1263}{|}^{2}>$ 0.999, Figure 3c), the proton transfers from the hydroxyl oxygen to the nitrogen atom (Figure 3a). Because this enol to keto transformation is accompanied by a 1.4 eV red-shift of the S_1_–S_0_ energy gap (Figure 1d), HBQ becomes resonant with the MeB molecules as well as with the cavity modes (Figure 2), and enters the dark state manifold around 10 fs after excitation.

Because after the reaction, HBQ couples collectively with the MeB molecules to the cavity modes, population transfers from HBQ into the cavity modes (Figure S7), and starts propagating at the central group velocity of these modes. Although the propagation is ballistic, this motion is interrupted by population transfer from the propagating cavity modes into the strongly coupled MeB excitons (Figure 3d), which are stationary. Because this exchange is reversible (Figure S6), wavepacket propagation is diffusive rather than ballistic, as indicated by a linear dependence of the mean squared displacement on time (Figure 3e; for ballistic propagation, the MSD_exc_ would be quadratic).

Because the excitation propagates along the molecules via the lossy cavity modes (Figure 3b), radiative decay competes with population transfer into the molecular states, which reduces the transport efficiency. To understand the influence of such losses, we repeated the simulations in a higher-*Q* cavity with a lifetime of 100 fs, as well as in an ideal cavity with an infinite lifetime. The results of these additional simulations, summarized in Figures S10 and S11, suggest that increasing the cavity *Q*-factor can significantly enhance the transport, in line with previous experiments [14] and simulations [35]. While the mechanism remains the same, the increase in the duration of the ballistic phases between the transfers from and into the molecular states, enhances the diffusion constant.

The initial structures for our simulations were sampled from equilibrium QM/MM trajectories at 300 K (SM) and therefore can capture the heterogeneity as indicated by the absorption line-widths of the molecules in Figure 1e. Because of such structural disorder, the ESIPT reaction rates span a distribution. To confirm that the proton transfer in HBQ is required to initiate the polariton-mediated exciton transport process, we show in Figure S8 (SM) that for a system in which the ESIPT is delayed, also the transport starts at a later point in time, and that this time point coincides with the formation of the HBQ photo-product.

To provide further evidence that the photo-chemical reaction is essential for initiating transport, we also performed a simulation in which the bond distance between the oxygen and proton in HBQ is constrained [71]. Because with such constraint the proton cannot transfer, no photo-product that is resonant with the cavity can form. Therefore, population transfer is suppressed and exciton transport is not observed (Figure S9).

Because in our simulations, there are no restrictions on where the molecules are positioned, we could build a system in which HBQ was dissolved in cyclohexane but MeB in water. A practical realization, however, would likely require that both molecules are miscible within the same solvent or polymer material. While a wide variety of water-soluble photoacids [72], or other candidates for the photo-chemical initiation step are available, such compounds would not only require a sufficiently high absorptivity in the transparency windows of the cavity, but also a highly fluorescent photo-product, which itself must be formed with high quantum-yield.

Alternatively, if in addition to a bright S_1_ electronic state, the strongly-coupled dye also has higher-energy electronic states that absorb within the transparency window of the cavity mirrors, polariton transport could be initiated via internal conversion from such higher-energy excited state into the S_1_ state [73]. Indeed, laser excitation into a higher-energy electronic state has been used in previous experiments on polariton transport [8], [9], [13], [16]. However, for this mechanism to operate also under incoherent excitation conditions, the higher-energy transition has to be sufficiently strong, which could limit the selection of suitable molecules. Furthermore, if the transparency window is provided by a higher-order cavity mode, as in Figure S4 in SM, the higher-energy electronic transition of a single species can also strongly couple to the cavity and form delocalized polaritons with a rapid radiative decay channel that competes with the internal conversion process into a localized S_1_ state. As initiating transport with a photo-chemical reaction does not require that the photo-reactive molecules are strongly coupled to the cavity, these molecules can be introduced at low concentration, potentially even at specific locations inside the cavity system, which might offer further advantages for practical applications of polariton-enhanced exciton transport.

Although at the level of theory employed in our simulations, the emission maximum of the HBQ photo-product matches the absorption maximum of the strongly coupled MeB acceptors, such matching is not a strict requirement. Instead, overlap with the lower polaritonic branch is sufficient, which can be controlled further by tuning the cavity resonance via the distance between the mirrors, or by tuning the coupling strength via the concentration of the acceptor dye. Indeed, as we show in the SM, adding a (artificial) blue-shift of 250 meV to the acceptor and the fundamental cavity mode, such that the emission maximum of HBQ overlaps with the LP branch rather than with the MeB absorption (Figure S12), does not affect the transport (Figure S13). The latter observation is consistent with experiments of Akselrod and co-workers who used emission from an uncoupled dye that is resonant with the lower polariton of a strongly coupled dye, to pump that lower polariton branch and trigger condensation [74].

The current limitations on computer hard- and software restrict the number of molecules we can model in our atomistic MD simulations to a few thousand, which is a few orders of magnitude smaller than the number of molecules in a real Fabry–Pérot cavity (*i.e.*, 10^6^–10^8^) [29], [75]. To reach the strong coupling regime under these conditions, and achieve a Rabi splitting of 282 meV, we therefore used a vacuum field strength that is significantly larger than in experiments. Because the light–matter coupling that drives the population transfer between dark and bright states, is inversely proportional to the number of molecules, *N* [29], [76], [77], the rate of population transfer also scales as 1/*N*, and is thus much faster in our simulation than in experiment. In previous work [34], we had therefore investigated how the transport depends on *N*, and found that the propagation velocity scales as 1/*N* as well. Thus, even in the limit of realistic *N*, we would still expect polariton-mediated exciton transport to exceed the intrinsic exciton diffusion process in organic materials, in line with experimental observations [9].

## 4 Conclusions

To summarize, the results of our MD simulations suggest that long-range polariton-mediated exciton transport can be induced with an excited-state proton transfer reaction. While the excitation scheme proposed here resembles the off-resonant laser excitation conditions employed in previous experiments on polariton transport [8], [9], [13], [16], the absorption cross-section of HBQ should be high enough to initiate the propagation with incoherent light, in particular for a cavity with a thin silver top mirror, which is more than 50 % transparent at the required wave length (Figure S4, SM).

## Supplementary Material

Supplementary Material Details
