# Supplementary material for: Photochemical initiation of polariton-mediated exciton propagation
Source: Nanophotonics. 2024 Jan 16;13(14):2687–94. doi: 10.1515/nanoph-2023-0684 (PMC11636319; doi:10.1515/nanoph-2023-0684)
Supplement: Supplementary file 1 — Supplementary Material Details [file j_nanoph-2023-0684_suppl_001.pdf]

Supplementary Material

for

Photochemical initiation of polariton-mediated exciton propagation

Ilia Sokolovskii and Gerrit Groenhof*

Nanoscience Center and Department of Chemistry, University of Jyväskylä, P.O. Box 35, 40014 Jyväskylä, Finland.

E-mail: gerrit.x.groenhof@jyu.fi

Contents

1 Molecular Dynamics Simulation Model **4**

1.1 Multiscale Tavis-Cummings Hamiltonian 4

1.2 One-dimensional Periodic Cavity 5

1.3 Ehrenfest dynamics 8

2 Simulation details **9**

2.1 HBQ model 9

2.2 Methylene Blue model 10

2.3 HBQ/MeB cavity simulations 13

3 Simulation Analysis **13**

3.1 Monitoring exciton dynamics in the strong coupling regime 13

3.2 Photo-Absorption Spectra 14

3.2.1 Molecular absorption spectra 14

3.2.2 Cavity absorption spectra 15

4 Additional Results **17**

4.1 Transparency of cavity mirrors 17

4.2 Potential energy surface of HBQ 18

4.3 Population exchange between diabatic states 19

4.4 Cavity Mode Contribution 20

4.5 Delayed exciton transport when HBQ reacts later 21

4.6 Simulations with constrained O-H bond in HBQ 22

4.7 Effect of cavity decay 24

4.8 Blue-shifted polariton branches 27

5 Animations **29**

6 References 29

1 Molecular Dynamics Simulation Model

1.1 Multiscale Tavis-Cummings Hamiltonian

Within the single-excitation subspace, which is probed experimentally under weak driving conditions, and employing the rotating wave approximation (RWA), which is valid for light-matter coupling strengths below 10% of the material excitation energy,[1] the interaction between N molecules and n_{mode} confined light modes in a one-dimensional (1D) Fabry-Pérot cavity (Figure S1) is modelled with the multi-scale Molecular Dynamics (MD) extension of the traditional Tavis-Cummings model of quantum optics:[2–5]

$$\begin{aligned}\hat{H}^{\text{TC}} \quad = \quad & \sum_j^N h\nu_j(\mathbf{R}_j)\hat{\sigma}_j^+\hat{\sigma}_j^- + \sum_{k_z}^{n_{\text{mode}}} \hbar\omega_{\text{cav}}(k_z)\hat{a}_{k_z}^\dagger \hat{a}_{k_z} + \\ & \sum_j^N \sum_{k_z}^{n_{\text{mode}}} \hbar g_j(k_z)\left(\hat{\sigma}_j^+ \hat{a}_{k_z} e^{ik_z z_j} + \hat{\sigma}_j^- \hat{a}_{k_z}^\dagger e^{-ik_z z_j}\right) + \\ & \sum_i^N V_{\text{S}_0}^{\text{mol}}(\mathbf{R}_i)\end{aligned} \tag{1}$$

Here, $\hat{\sigma}_j^+$ ($\hat{\sigma}_j^-$) is the operator that excites (de-excites) molecule j from the electronic ground (excited) state $|\text{S}_0^j(\mathbf{R}_j)\rangle$ ($|\text{S}_1^j(\mathbf{R}_j)\rangle$) to the electronic excited (ground) state $|\text{S}_1^j(\mathbf{R}_j)\rangle$ ($|\text{S}_0^j(\mathbf{R}_j)\rangle$); $\mathbf{R}_j$ is the vector of the Cartesian coordinates of all atoms in molecule j, centered at z_j; $\hat{a}_{k_z}$ ($\hat{a}_{k_z}^\dagger$) is the annihilation (creation) operator of an excitation of a cavity mode with wave-vector k_z; $h\nu_j(\mathbf{R}_j)$ is the excitation energy of molecule j, defined as:

$$h\nu_j(\mathbf{R}_j) = V_{\text{S}_1}^{\text{mol}}(\mathbf{R}_j) - V_{\text{S}_0}^{\text{mol}}(\mathbf{R}_j) \tag{2}$$

with $V_{\text{S}_0}^{\text{mol}}(\mathbf{R}_j)$ and $V_{\text{S}_1}^{\text{mol}}(\mathbf{R}_j)$ the adiabatic potential energy surfaces (PESs) of molecule j in the electronic ground (S_0) and excited (S_1) state, respectively. The last term in Equation 1 is the total potential energy of the system in the absolute ground state (*i.e.*, with no excitations in neither the molecules nor the cavity modes), defined as the sum of the ground-state potential energies of all

molecules in the cavity. The $V^{\text{mol}}_{\text{S}_0}(\mathbf{R}_j)$ and $V^{\text{mol}}_{\text{S}_1}(\mathbf{R}_j)$ adiabatic PESs are modelled at the hybrid quantum mechanics / molecular mechanics (QM/MM) level of theory.[6,7]

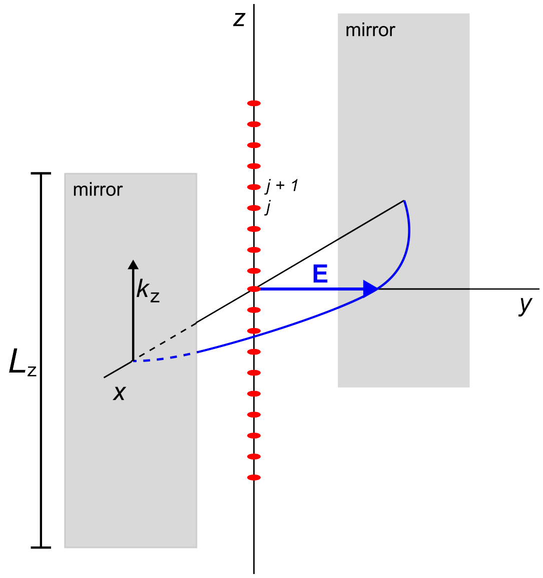

Figure S1: One-dimensional (1D) Fabry-Pérot micro-cavity model. Two reflecting mirrors located at $-\frac{1}{2}x$ and $\frac{1}{2}x$, confine light modes along this direction, while free propagation along the z direction is possible for plane waves with in-plane momentum k_z and energy $\hbar\omega_{\text{cav}}(k_z)$. The vacuum field vector (red) points along the y-axis, reaching a maximum amplitude at $x = 0$ where the N molecules (magenta ellipses) are placed, distributed along the z-axis at positions z_j with $1 \leq j \leq N$.

The third term in Equation 1 describes the light-matter interaction within the dipolar approximation through $g_j(k_z)$:

$$g_j(k_z) = -\boldsymbol{\mu}^{\text{TDM}}_j(\mathbf{R}_j) \cdot \mathbf{u}_{\text{cav}} \sqrt{\frac{\hbar\omega_{\text{cav}}(k_z)}{2\epsilon_0 V_{\text{cav}}}} \tag{3}$$

where $\boldsymbol{\mu}^{\text{TDM}}_j(\mathbf{R}_j)$ is the transition dipole moment of molecule j that depends on the molecular geometry ($\mathbf{R}_j$); $\mathbf{u}_{\text{cav}}$ the unit vector in the direction of the electric component of cavity vacuum field (*i.e.*, $|\mathbf{E}| = \sqrt{\hbar\omega_{\text{cav}}(k_z)/2\epsilon_0 V_{\text{cav}}}$), here along the y-direction (Figure S1); ϵ_0 the vacuum permittivity; and V_{cav} the cavity mode volume.

1.2 One-dimensional Periodic Cavity

Following Michetti and La Rocca,[4] we impose periodic boundary conditions in the z-direction of our cavity, and thus restrict the wave vectors, k_z, to discrete values: $k_{z,p} = 2\pi p/L_z$ with $p \in \mathbb{Z}$ and

L_z the length of the 1D cavity. With this approximation the molecular Tavis-Cummings Hamiltonian in Equation 1 can be represented as an $(N + n_{\text{mode}})$ by $(N + n_{\text{mode}})$ matrix with four blocks:[5]

$$\mathbf{H}^{\text{TC}} = \begin{pmatrix} \mathbf{H}^{\text{mol}} & \mathbf{H}^{\text{int}} \\ \mathbf{H}^{\text{int}\dagger} & \mathbf{H}^{\text{cav}} \end{pmatrix} \tag{4}$$

We compute the elements of this matrix in the product basis of adiabatic molecular states times cavity mode excitations:

$$\begin{aligned} |\phi_j\rangle &= \hat{\sigma}_j^+ |\text{S}_0^1 \text{S}_0^2..\text{S}_0^{N-1}\text{S}_0^N\rangle \otimes |00..0\rangle \\ &= \hat{\sigma}_j^+ |\Pi_i^N \text{S}_0^i\rangle \otimes |\Pi_k^{n_{\text{mode}}} 0_k\rangle \\ &= \hat{\sigma}_j^+ |\phi_0\rangle \end{aligned} \tag{5}$$

for $1 \leq j \leq N$, and

$$\begin{aligned} |\phi_{j>N}\rangle &= \hat{a}_{j-N}^\dagger |\text{S}_0^1 \text{S}_0^2..\text{S}_0^{N-1}\text{S}_0^N\rangle \otimes |00..0\rangle \\ &= \hat{a}_{j-N}^\dagger |\Pi_i^N \text{S}_0^i\rangle \otimes |\Pi_k^{n_{\text{mode}}} 0_k\rangle \\ &= \hat{a}_{j-N}^\dagger |\phi_0\rangle \end{aligned} \tag{6}$$

for $N < j \leq N + n_{\text{mode}}$. In these expressions $|00..0\rangle$ indicates that the Fock states for all n_{mode} cavity modes are empty. The basis state $|\phi_0\rangle$ is the ground state of the molecule-cavity system with no excitations of neither the molecules nor cavity modes:

$$|\phi_0\rangle = |\text{S}_0^1 \text{S}_0^2..\text{S}_0^{N-1}\text{S}_0^N\rangle \otimes |00..0\rangle = |\Pi_i^N \text{S}_0^i\rangle \otimes |\Pi_k^{n_{\text{mode}}} 0_k\rangle \tag{7}$$

The upper left block, $\mathbf{H}^{\text{mol}}$, is an $N \times N$ matrix containing the single-photon excitations of the molecules. Because we neglect direct excitonic interactions between molecules, this block is

diagonal, with elements labeled by the molecule indices j:

$$H^{\text{mol}}_{j,j} = \langle \phi_0 | \hat{\sigma}_j \hat{H}^{\text{TC}} \hat{\sigma}^+_j | \phi_0 \rangle \tag{8}$$

for $1 \leq j \leq N$. Each matrix element of $\mathbf{H}^{\text{mol}}$ thus represents the potential energy of a molecule, j, in the electronic excited state $|\text{S}^j_1(\mathbf{R}_j)\rangle$ while all other molecules, $i \neq j$, are in the electronic ground state $|\text{S}^i_0(\mathbf{R}_i)\rangle$:

$$H^{\text{mol}}_{j,j} = V^{\text{mol}}_{\text{S}_1}(\mathbf{R}_j) + \sum_{i \neq j}^{N} V^{\text{mol}}_{\text{S}_0}(\mathbf{R}_i) \tag{9}$$

The lower right block, $\mathbf{H}^{\text{cav}}$, is an $n_{\text{mode}} \times n_{\text{mode}}$ matrix (with $n_{\text{mode}} = n_{\text{max}} - n_{\text{min}} + 1$) containing the single-photon excitations of the cavity modes, and is also diagonal:

$$H^{\text{cav}}_{p,p} = \langle \phi_0 | \hat{a}_p \hat{H}^{\text{TC}} \hat{a}^{\dagger}_p | \phi_0 \rangle \tag{10}$$

for $n_{\text{min}} \leq p \leq n_{\text{max}}$. Here, $\hat{a}^{\dagger}_p$ excites cavity mode p with wave-vector $k_{z,p} = 2\pi p / L_z$. In these matrix elements, all molecules are in the electronic ground state (S_0). The energy is therefore the sum of the cavity energy at $k_{z,p}$, and the molecular ground state energies:

$$H^{\text{cav}}_{p,p} = \hbar \omega_{\text{cav}}(2\pi p / L_z) + \sum_{j}^{N} V^{\text{mol}}_{\text{S}_0}(\mathbf{R}_j) \tag{11}$$

where, $\omega_{\text{cav}}(k_{z,p})$ is the cavity dispersion (dashed curve in Figure 1f, main text):

$$\omega_{\text{cav}}(k_{z,p}) = \sqrt{\omega_0^2 + c^2 k^2_{z,p} / n^2} \tag{12}$$

with $\hbar\omega_0$ the energy at $k_0 = 0$, n the refractive index of the medium and c the speed of light in vacuum.

The two $N \times n_{\text{mode}}$ off-diagonal blocks $\mathbf{H}^{\text{int}}$ and $\mathbf{H}^{\text{int}\dagger}$ in the multi-mode Tavis-Cummings Hamiltonian (Equation 4) model the light-matter interactions between the molecules and the cavity modes. Within the long-wavelength approximation these matrix elements can be approximated by

the overlap between the transition dipole moment of molecule j and the transverse electric field of cavity mode p at the geometric center z_j of that molecule:

$$\begin{aligned} H^{\text{int}}_{j,p} &= -\boldsymbol{\mu}^{\text{TDM}}_j(\mathbf{R}_j)\cdot\mathbf{u}_{\text{cav}}\sqrt{\frac{\hbar\omega_{\text{cav}}(2\pi p/L_z)}{2\epsilon_0 V_{\text{cav}}}}\langle\phi_0|\hat{\sigma}_j\hat{\sigma}^+_j\hat{a}_p e^{i2\pi p z_j/L_z}\hat{a}^\dagger_p|\phi_0\rangle \\ &= -\boldsymbol{\mu}^{\text{TDM}}_j(\mathbf{R}_j)\cdot\mathbf{u}_{\text{cav}}\sqrt{\frac{\hbar\omega_{\text{cav}}(2\pi p/L_z)}{2\epsilon_0 V_{\text{cav}}}}e^{i2\pi p z_j/L_z} \end{aligned} \quad (13)$$

for $1 \leq j \leq N$ and $n_{\text{min}} \leq p \leq n_{\text{max}}$.

1.3 Ehrenfest dynamics

In our simulations classical trajectories evolve under the influence of the expectation value of forces with respect to the polaritonic wave function,[8] while the polaritonic wave function evolves along with the classical degrees of freedom. By expanding the total wave function as a linear combination of the *time-independent* diabatic light-matter states (Equations 5 and 6):

$$|\Psi(t)\rangle = \sum_j^{N+n_{\text{modes}}} |\phi_j\rangle c_j(t) \quad (14)$$

the evolution of the *time-dependent* diabatic expansion coefficients, $c_j(t)$, is obtained by numerically integrating the Schrödinger equation over discrete time intervals, Δt.

$$\mathbf{c}(t+\Delta t) = \mathbf{P}^{\text{dia}}\mathbf{c}(t) \quad (15)$$

Here, $\mathbf{c}(t)$ is a vector containing the diabatic expansion coefficients $c_j(t)$ and $\mathbf{P}^{\text{dia}}$ the propagator in the diabatic basis

$$\mathbf{P}^{\text{dia}} = \exp\left[-i\left(\mathbf{H}^{\text{TC}}(t+\Delta t) + \mathbf{H}^{\text{TC}}(t) - i\hbar\boldsymbol{\gamma}\right)\Delta t/2\hbar\right] \quad (16)$$

To account for the losses due to photon-leakage through the imperfect cavity mirrors, we add the decay rates of the cavity modes to the Tavis-Cummings Hamiltonian, here represented as a diagonal matrix, $\boldsymbol{\gamma}$, with the cavity mode decay rates γ_k as elements.[9–12] Because of these decay terms, the norm of the total wave function, $|\Psi(t)|^2 = \sum_j^{N+n_{\text{modes}}} |c_j|^2$, is not conserved but decreases due to the losses.

2 Simulation details

2.1 HBQ model

The Gromos-2016H66 force field was used to model the interactions, because it contains a validated model for cyclohexane,[13] which was used as the solvent for HBQ in our simulations. In this united-atom representation of cyclohexane none of the atoms carries a partial charge. Because HBQ was kept frozen during the solvent equilibration and modeled at the QM level in all other simulations, there was no need for assigning partial charges to the HBQ atoms. The Gromos96 atom-types used for HBQ to model the Van der Waals interactions, described by the Lennard-Jones potential, between HBQ and the cyclohexane molecules, are `HC` for all aromatic hydrogen atoms, `C` for all carbon atoms, `NR` for the aromatic nitrogen atom, `OA` for the hydroxyl oxygen atom and `H` for the hydroxyl proton.

One HBQ molecule was geometry-optimized and placed inside a rectangular box that was subsequently filled with 402 cyclohexane molecules. The simulation box, which thus contained 2436 atoms, was equilibrated for 100 ns. During equilibration, the coordinates of the HBQ atoms were kept fixed. The LINCS algorithm was used to constrain bond lengths in cyclohexane,[14] enabling a time step of 2 fs. Temperature and pressure were maintained at 300 K and 1 atmosphere by means of weak-coupling to an external bath (τ_T = 0.1 ps, τ_P = 1 ps).[15] The Lennard-Jones potential was truncated at 1.4 nm.

Snapshots were extracted from the equilibration trajectory and further equilibrated for 50 ps at the QM/MM level with a time step of 1 fs. In these QM/MM simulations HBQ was modelled

with density functional theory (DFT), using the CAM-B3LYP functional,[16–18] in combination with a 3-21G basis set.[19] The cyclohexane solvent was described with the 2016H66 parameter set of the Gromos96 force field.[13] The QM subsystem was mechanically embedded within the MM subsystem. Because cyclohexane atoms are uncharged, the interactions between the QM and MM regions were modeled with Lennard-Jones potentials only. We used time-dependent DFT (TD-DFT),[20] within the Tamm-Dancoff approximation (TDA),[21] in combination with the CAM-B3LYP functional and the 3-21G basis set, to model the singlet excited electronic (S_1) state of HBQ. All QM/MM simulations of HBQ outside of the cavity were performed with GROMACS version 4.5.3,[22] using the QM/MM interface to TeraChem version 1.93.[23,24]

2.2 Methylene Blue model

The Amber03 force field was used to model the interactions between Methylene Blue (MeB) and the water solvent, which was described with the TIP3P water model.[25] Atom types and partial charges for the MeB atoms (Figure S2) are listed in Table S1. The partial charges of the atoms were derived following the procedure recommended for the Amber03 force field.[26,27] First, the geometry of MeB was minimized at the HF/6-31G** level of *ab initio* theory, using the IEFPCM continuum solvent model with a relative dielectric constant of 4.0.[28] After the geometry optimizations, the electrostatic potential at 10 concentric layers of 17 points per unit area around each atom was evaluated using the electron density calculated at the B3LYP/cc-pVTZ level of DFT theory,[16] again using the IEFPCM continuum solvent model with a relative dielectric constant of 4.0.[28] The atomic charges were obtained by performing a two-stage RESP fit to the electrostatic potential,[27] the first without symmetry constraints, and the second with symmetry constraints on chemically equivalent atoms.

A single MeB molecule was geometry-optimized at the B97/3-21G level of theory and placed at the center of a rectangular box that was filled with 2031 TIP3P water molecules.[25] A 1.0 nm cut-off was used for the Van der Waals' interactions, which were modeled with Lennard-Jones potentials, while the Coulomb interactions were computed with the smooth particle mesh Ewald method,[29]

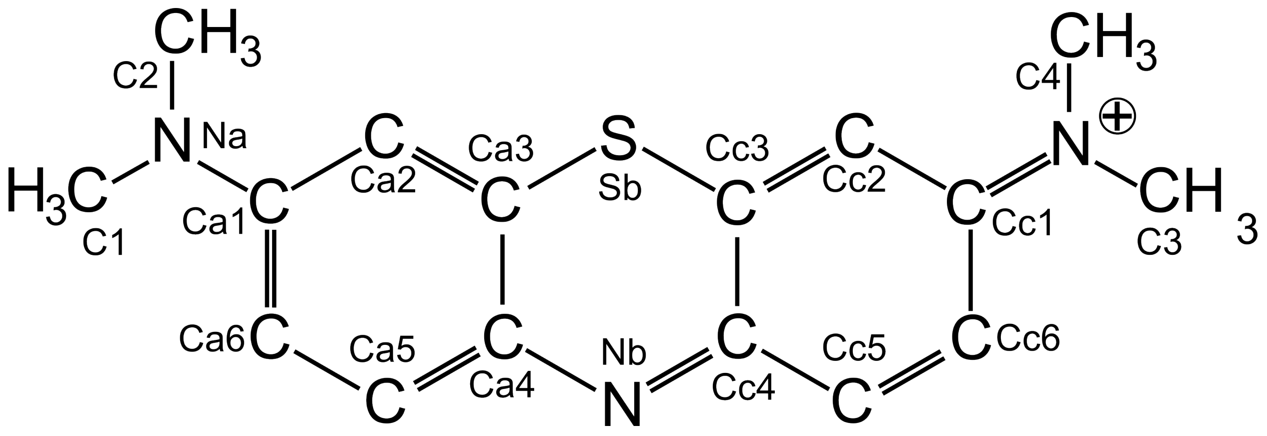

Figure S2: Structure and atom names of Methylene Blue. Not all hydrogens are shown, but their names take the name of the carbon atom plus an integer, *e.g.*, HA2 is attached to CA2 and H21, H22 and H23 are attached to carbon C2.

using a 1.0 nm real space cut-off and a grid spacing of 0.12 nm and a relative tolerance at the real space cut-off of 10^{-5}. The simulation box, containing 6,131 atoms, was equilibrated for 1 ns at the force field level of theory. During this equilibration, the coordinates of the MeB atoms were kept fixed. The temperature was maintained at 300 K with the v-rescale thermostat,[30] while the pressure was kept constant at 1 atmosphere using the Berendsen isotropic pressure coupling algorithm,[15] with a time constant of 1 ps. The SETTLE algorithm was applied to constrain the internal degrees of freedom of water molecules,[31] enabling a time step of 2 fs in the classical MD simulations.

After equilibration at the force field (MM) level, the system was further equilibrated at the QM/MM level for 10 ps. The time step was reduced to 1 fs. The Methylene Blue molecule was modelled at the DFT level, using the B97 functional,[32] in combination with the 3-21G basis set.[19] The water solvent was modelled with the TIP3P force field.[25] The QM system experienced the Coulomb field of all MM atoms within a 1.0 nm cut-off sphere and Lennard-Jones interactions between MM and QM atoms were added. The singlet electronic excited state (S_1) was modeled with TD-DFT,[20] using the B97 functional in combination with the 3-21G basis set for the QM region (*i.e.,* TD-B97/3-21G),[19]. At this level of QM/MM theory, the excitation energy is 2.5 eV. The overestimation of the vertical excitation energy is due to the limited accuracy of the employed level of theory, but was compensated by adding an off-set to the cavity resonance energy (see subsection 2.3). The QM/MM simulations were performed with GROMACS version 4.5.3,[22]

Table S1: Amber03 atomtypes and partial charges for Methylene Blue. Atom names are defined in Figure S2.

name	type	charge (e)
Ca1	CA	0.054312
Na	NA	0.154719
C1	CT	-0.284923
H11	H1	0.128201
H12	H1	0.128201
H13	H1	0.128201
C2	CT	-0.275632
H21	H1	0.127095
H22	H1	0.127095
H23	H1	0.127095
Ca2	CA	-0.205067
Ha2	HA	0.181067
Ca3	C	-0.101342
Ca4	CA	0.624779
Ca5	CA	-0.286158
Ha5	HA	0.187369
Ca6	CA	-0.141958
Ha6	HA	0.158598
Nb	NB	-0.712421
Sb	S	0.049117
Cc1	CA	0.054312
Nc	NA	0.154719
C3	CT	-0.284923
H31	H1	0.128201
H32	H1	0.128201
H33	H1	0.128201
C4	CT	-0.275632
H41	H1	0.127095
H42	H1	0.127095
H43	H1	0.127095
Cc2	CA	-0.205067
Hc2	HA	0.181067
Cc3	C	-0.101342
Cc4	CA	0.624779
Cc5	CA	-0.286158
Hc5	HA	0.187369
Cc6	CA	-0.141958
Hc6	HA	0.158598

interfaced to TeraChem version 1.93.[23,24]

2.3 HBQ/MeB cavity simulations

From the HBQ QM/MM trajectory, we selected single HBQ snapshots, which we combined with 1023 Methylene Blue molecules from the MeB QM/MM trajectory. These 1024 molecules, including their solvent environments, were placed at equal inter-molecular separations on the z-axis of a 1D,[4] 50 µm long, symmetric optical Fabry-Pérot micro-cavity, illustrated in Figure S1. The dispersion of this cavity was modelled with 239 discrete modes (*i.e.*, $k_{z,p} = 2\pi p/L_z$ with $-119 \leq p \leq 119$ and $L_z = 50$ µm). The micro-cavity was red-detuned with respect to the Methylene Blue absorption maximum, which is 2.5 eV at the TD-B97/3-21G//Amber03 level of theory used in this work, such that the energy of the fundamental cavity mode at zero incidence angle ($k_0 = 0$) was $\hbar\omega_0 = 2.18$ eV. To maximize the collective light-matter coupling strength, the transition dipole moments of all molecules were aligned to the vacuum field at the start of the simulation. With a vacuum field strength of $\mathbf{E}_y = 0.00004$ (0.21 MVcm^{-1}) this configuration yields a Rabi splitting of $\hbar\Omega^{\text{Rabi}} = 323$ meV. The resulting polariton dispersion, as well as the group velocity of lower and upper polaritons are shown in Figure S3.

3 Simulation Analysis

3.1 Monitoring exciton dynamics in the strong coupling regime

To track the propagation of the excitonic wavepacket, we plotted the probability density of the excitonic contributions to the total time-dependent wave function $|\Psi(t)|^2$ at the positions of the molecules, z_j, as a function of time. We thus represent the density as a *discrete* distribution at grid points that correspond to the molecular positions, rather than a continuous distribution. The amplitude of the excitonic wave function, $|\Psi_{\text{exc}}(t)\rangle$, at position z_j in the 1D cavity (with $z_j = (j-1)L_z/N$ for $1 \leq j \leq N$) is obtained by projecting the excitonic basis state in which

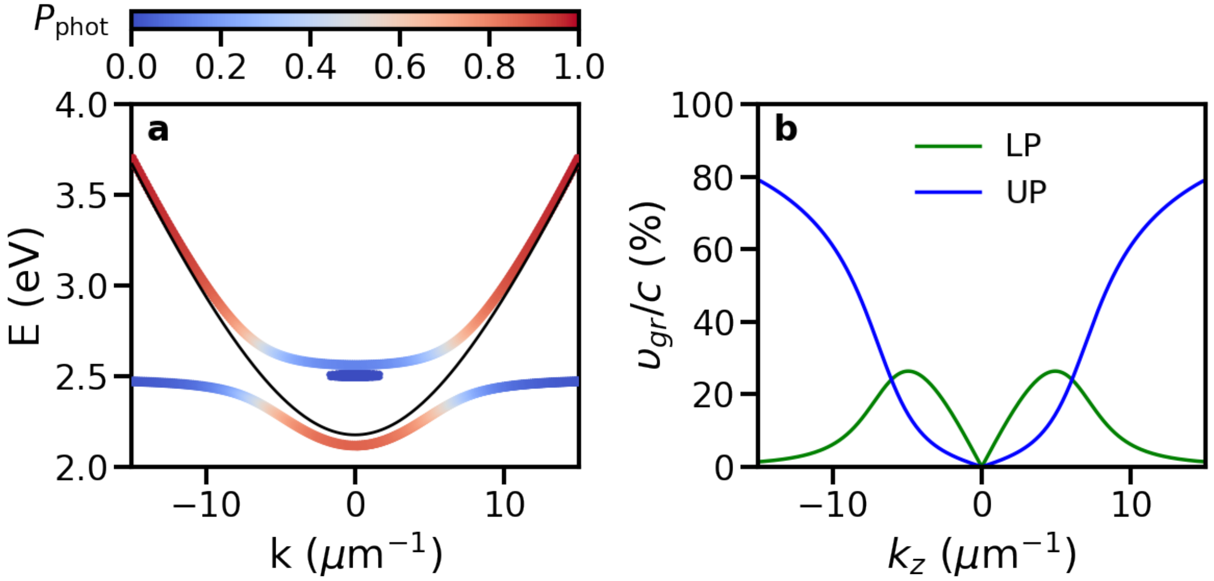

Figure S3: Panel **a**: Polariton dispersion of our 1D model cavity. The total contribution of all cavity modes to each polaritonic state (P_{phot}) is indicated by colors. Dispersion of the empty Fabry-Pérot cavity is shown in black. Panel **b**: Group velocity, $\upsilon_{\text{gr}} = \partial\omega_{\text{pol}}/\partial k_z$, of lower (green line) and upper (blue line) polaritons as a fraction of the speed of light, c.

molecule j at position z_j is excited (*i.e.*, $|\phi_j\rangle = \hat{\sigma}_j^+|\phi_0\rangle$, Equation 5) to the total wave function (Equation 14):

$$|\Psi_{\text{exc}}(z_j, t)\rangle = \hat{\sigma}_j^+|\phi_0\rangle\langle\phi_0|\hat{\sigma}_j|\Psi(t)\rangle = c_j(t) \tag{17}$$

where the $c_j(t)$ are the time-dependent expansion coefficients of the diabatic states, $|\phi_j\rangle$, in total wave function $|\Psi(t)\rangle$.

3.2 Photo-Absorption Spectra

3.2.1 Molecular absorption spectra

The absorption spectra of HBQ and MeB were computed from QM/MM trajectories in the ground (S_0) state. These spectra were constructed from the S_1-S_0 energy gaps in combination with the

transition dipole moments, through a super-position of Gaussian functions:[33]

$$I_{\text{mol}}^{\text{abs}}(E) \propto \sum_{i}^{s} \Delta E_i \exp\left[-\frac{(E-\Delta E_i)^2}{2\sigma^2}\right] |\boldsymbol{\mu}_i^{\text{TDM}}|^2 \tag{18}$$

where $I_{\text{mol}}^{\text{abs}}(E)$ is the intensity of the molecular absorption as a function of excitation energy (E), s the number of snapshots included in the analysis, ΔE_i the excitation energy in snapshot i and $\boldsymbol{\mu}_i^{\text{TDM}}$ the transition dipole moment of that excitation. A width of $\sigma = 0.05$ eV was used for the convolution.

The emission spectrum of HBQ was computed from a 10 ps QM/MM trajectory in the excited (S_1) state. The first 100 fs were discarded to restrict the sampling to the relaxed photo-product state. The spectra were composed as in Equation 18, using the S_1-S_0 energy gaps and transition dipole moments from the trajectory. As for the absorption spectra, a width of $\sigma = 0.05$ eV was used for the convolution.

3.2.2 Cavity absorption spectra

To compute the absorption spectrum of the molecule-cavity system as a function of energy and k_z-vector, we first diagonalized the Tavis-Cummings Hamiltonian, using molecular energies and transition dipole moments from the equilibrium QM/MM simulations, to obtain the $N + n_{\text{mode}}$ hybrid light-matter states $|\psi^m\rangle$:[4,34]

$$|\psi^m\rangle = \left(\sum_{j}^{N} \beta_j^m \hat{\sigma}_j^+ + \sum_{p}^{n_{\text{mode}}} \alpha_p^m \hat{a}_p^\dagger\right) |\phi_0\rangle \tag{19}$$

with eigenenergies E_m. The β_j^m and α_p^m expansion coefficients reflect the contribution of the molecular excitations ($|S_1^j(\mathbf{R}_j)\rangle$) and the cavity mode excitations ($|1_p\rangle$) to polariton $|\psi^m\rangle$.

Following Lidzey and coworkers,[35] we define the "visibility", I^m, of polaritonic state $|\psi^m\rangle$ as the total photonic contribution to that state (*i.e.*, $I^m \propto \sum_p^{n_{\max}} |\alpha_p^m|^2$). Thus, for each frame of the MeB QM/MM trajectory, the polaritonic states were computed and the energy gaps of these states

with respect to the overall ground state (*i.e.*, E^0, with all molecules in S_0, no photon in the cavity, Equation 7) were extracted for all cavity modes, p, and summed up into a superposition of Gaussian functions, weighted by $|\alpha_p^m|^2$:

$$I^{\text{abs}}(E, k_{z,p}) \propto \sum_t^s \left[\sum_m^{N+n_{\text{max}}+1} |\alpha_{p,t}^m|^2 \exp[-\frac{(E - \Delta E_t^m)^2}{2\sigma^2}] \right] \tag{20}$$

Here, $I^{\text{abs}}(E, k_{z,p})$ is the absorption intensity as a function of excitation energy E and in-plane momentum $k_{z,p} = 2\pi p/L_z$, s the number of trajectory frames included in the analysis, ΔE_t^m the excitation energy of polaritonic state m in frame t (*i.e.*, $\Delta E_t^m = E_t^m - E_t^0$) and $\alpha_{p,t}^m$ the expansion coefficient of cavity mode p in polaritonic state m in that frame (equation 19). A width of $\sigma = 0.05$ eV was used for the convolution.

4 Additional Results

4.1 Transparency of cavity mirrors

The reflectivity of a hypothetical Farby-Pérot micro-cavity composed of a 20 nm silver mirror on top of a 167 nm acrylic polymer layer and deposited on a silver substrate, was computed with the "Reflectance Calculator" of Filmetrics® (`https://www.filmetrics.com/reflectance-calculator`) and plotted as a function of wavelength (black) in Figure S4, together with the absorption spectra of HBQ in polymethyl-methacrylate (PMMA) (blue),[36] and of MeB in water (red). Because the first order cavity mode is resonant with the main absorption peak of MeB centered at 668 nm, the cavity is transparent around that wavelength. With a sufficiently high concentration of MeB in the polymer layer, this absorption peak will split into the LP and UP bands, separated by the Rabi splitting. Because the reflectivity of the top mirror is reduced by more than 50% at the absorption maxima of HBQ (375 nm and 360 nm), we anticipate that this mixed HBQ/MeB-cavity system will still have a significant cross-section for selectively exciting HBQ, and initiate the transport via the excited-state intra-molecular proton transfer process.

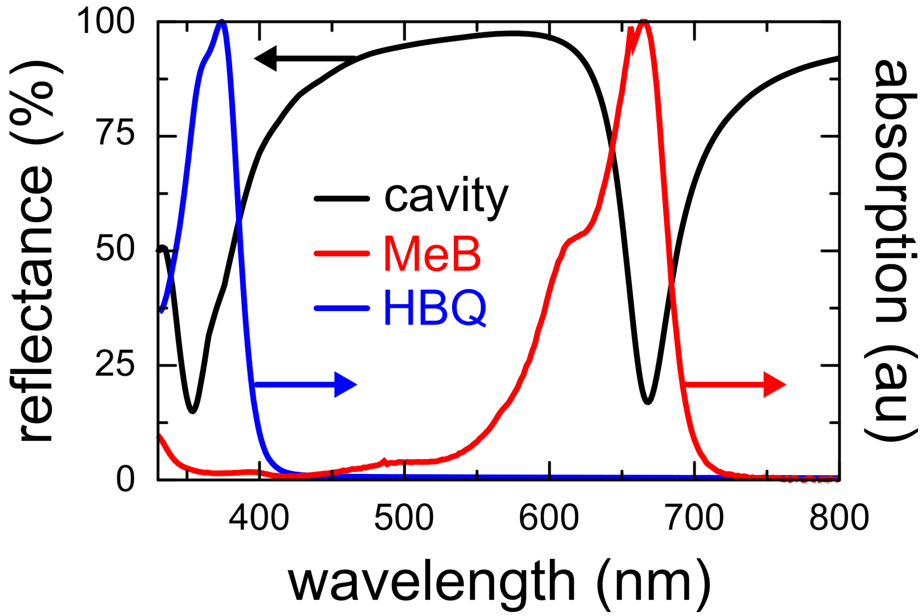

Figure S4: Calculated reflectance (black) spectrum of a Fabry-Pérot micro-cavity with a 20 nm silver mirror on top of a 167 nm polymer layer deposited on a silver substrate. Absorption spectra of MeB and HBQ are plotted in red and blue, respectively.

4.2 Potential energy surface of HBQ

In Figure S5 we compare the minimum energy potential energy profiles for the proton transfer reaction in the ground (S_0) and first excited electronic states (S_1) at the CAMB3LYP/3-21G,[18] and TPSSh/cc-pVDZ levels of theory.[37] These profiles were obtained by performing geometry optimizations on the S_1 potential energy surface at 33 equidistant points along the reaction coordinate, defined as the difference between the O-H and N-H distances (inset in Figure S5). The results of the relaxed scans suggest that the CAMB3LYP/3-21G model provides a potential energy surface on which proton transfer is barrierless in the excited state, in qualitative agreement with the calculations at the TPSSh/cc-pVDZ level of theory of Picconi,[38] that, in turn, are in good agreement with experiment.

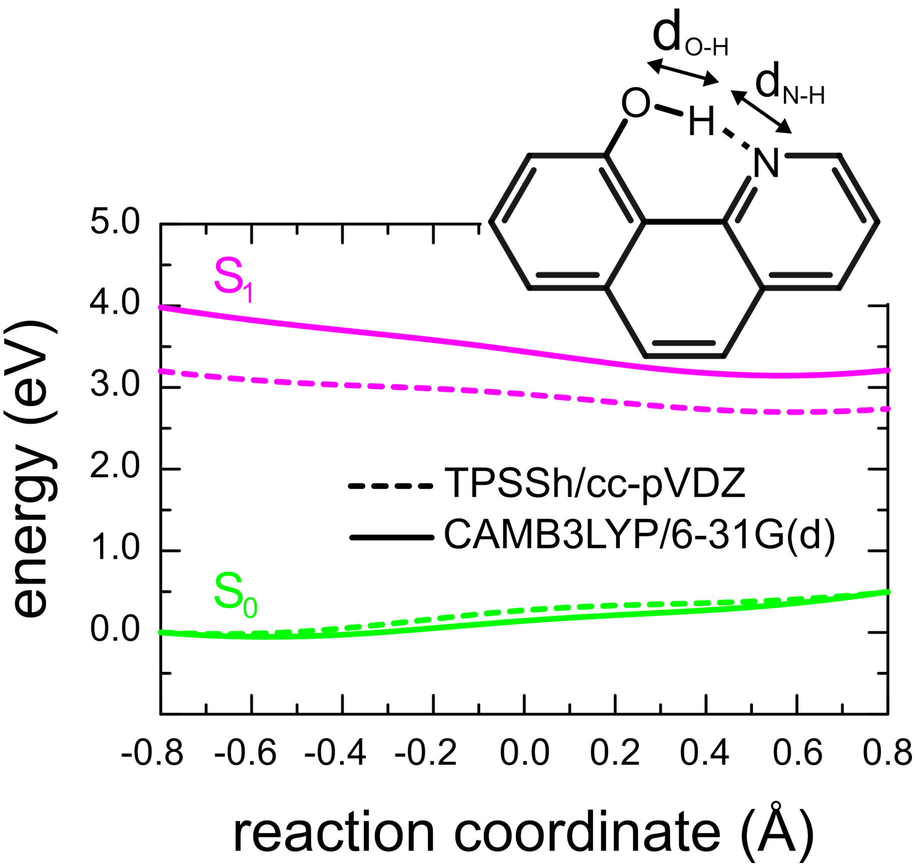

Figure S5: Minimum energy profile for excited-state intra-molecular proton transfer in HBQ as a function of the reaction coordinate defined as $d_{\text{O-H}} - d_{\text{N-H}}$ (inset) in the electronic ground state (S_0, green) and excited state (S_1, magenta) at the CAMB3LYP/3-21G (continuous lines) TPSSh/cc-pVDZ (dashed lines) levels of theory.

4.3 Population exchange between diabatic states

In Figure S6 we show the time evolution of the populations of the HBQ molecule, the MeB molecules and of the cavity modes excitations for two individual simulations in a lossless cavity (*i.e.*, $\gamma_{\text{cav}} = 0$ ps $^{-1}$). In both simulations, the population of the excited HBQ molecule is quickly transferred into the cavity modes, and from there into the MeB molecules. The out-of-phase fluctuations suggest that the population transfers reversibly between the strongly coupled MeB molecules and the cavity modes.

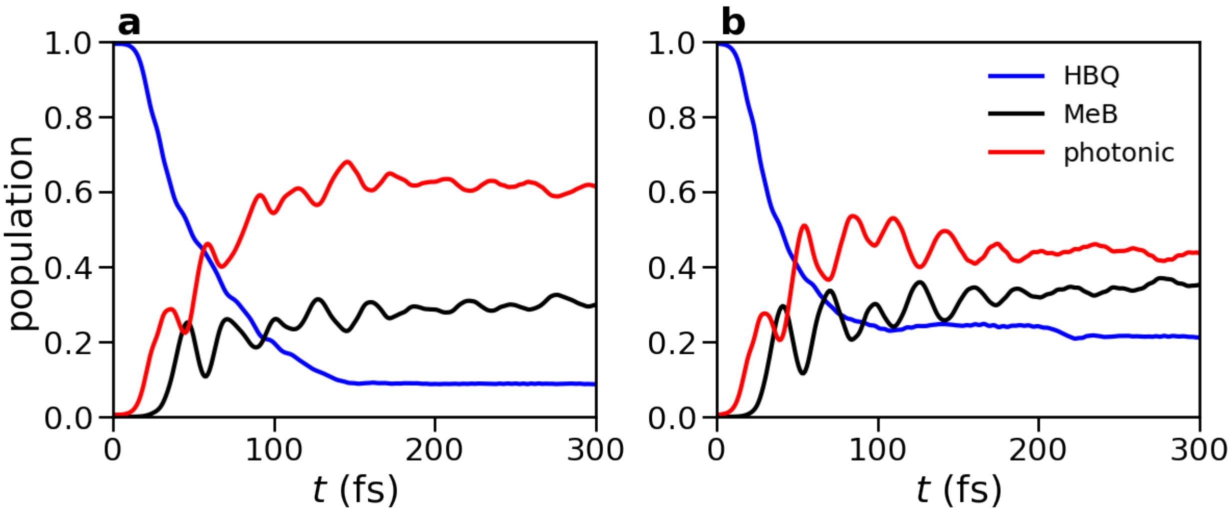

Figure S6: Populations of the HBQ molecule (blue), of all MeB molecules (black) and of all cavity mode excitations (red) as a function of time for two individual simulations in a cavity with a lifetime of $\gamma_{\text{cav}} = 0$ ps $^{-1}$.

4.4 Cavity Mode Contribution

To determine which cavity modes contribute to the excitation transport, we have calculated the time-average of the populations in all $n_{\rm mode} = 239$ cavity modes (*i.e.*, $\langle|c_p(t)|^2\rangle$ for $N < p \leq N + n_{\rm mode}$, Equation 2 in the main text) over the MD trajectories. As shown in Figure S7, the modes contributing most to polaritonic propagation, have energies that overlap with the MeB absorption spectrum, with a larger contribution on the blue side of that spectrum.

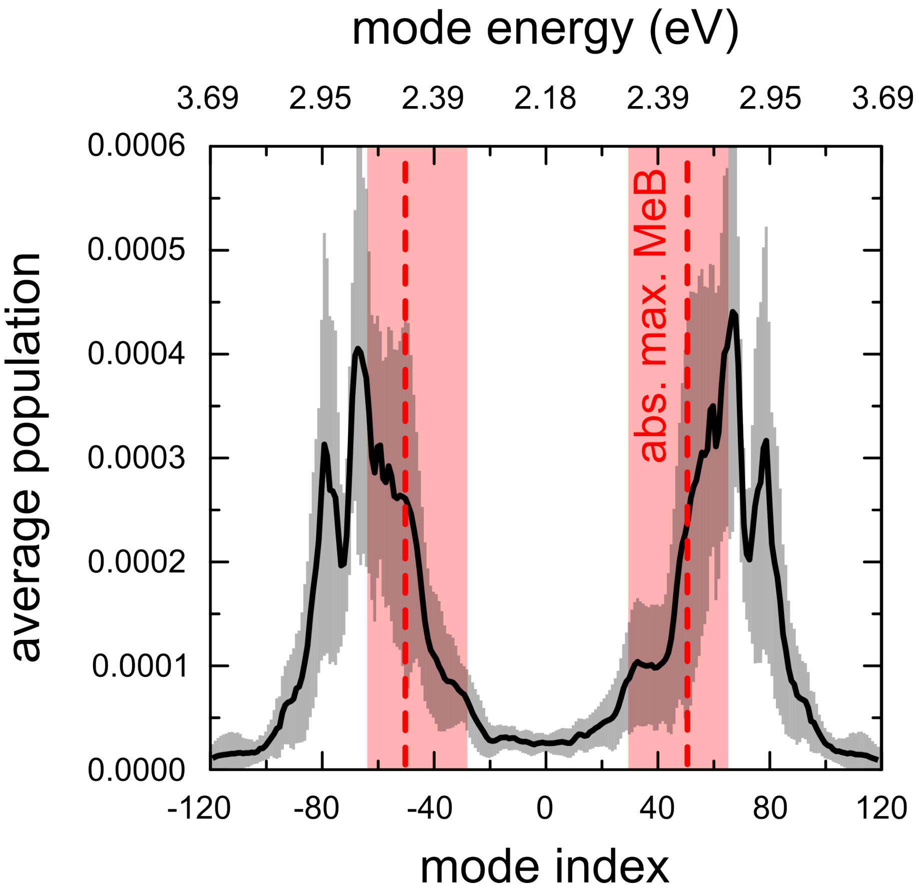

Figure S7: Time-averaged populations of the cavity modes, $\langle|c_p(t)|^2\rangle$. The black line shows the average over four MD trajectories, while the grey shaded area indicates the Root-mean-square deviation. The maximum and the full width at half maximum (FWHM) of the MeB absorption spectum are shown as the red dashed line and the red shaded area, respectively.

4.5 Delayed exciton transport when HBQ reacts later

In Figure S8 we show that when ESIPT occurs later, here 50 fs after photo-excitation, also the polariton-mediated exciton transport starts later.

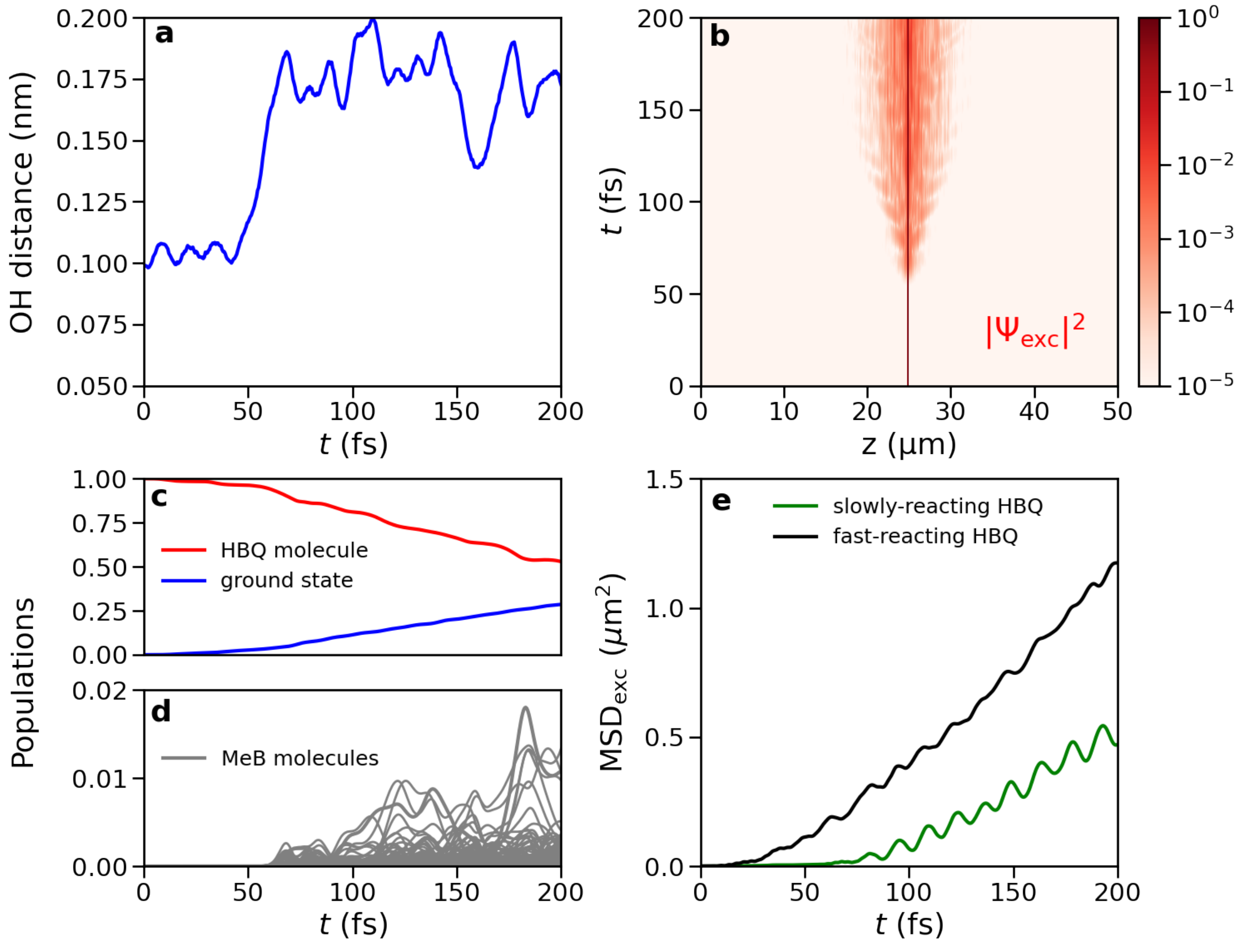

Figure S8: Delayed polariton-mediated exciton transport when the ESIPT in HBQ occurs later. Panel **a** shows the distance between the hydroxyl oxygen and proton, which we use as the reaction coordinate for the excited state proton transfer reaction (Figure S5), as a function of time after instantaneous excitation into the highest-energy eigenstate of the molecule-cavity system, which is dominated by the S_1 state of HBQ (*i.e.*, $|\beta_{\text{HBQ}}|^2 \approx 0.99$). Panel **b** shows the probability density of the excitonic part, $|\Psi_{\text{exc}}|^2$, of the total wavefunction as a function of the z-coordinate (horizontal axis) and time (vertical axis). Panels **c** and **d** show the contribution of the HBQ molecule (red) and Methylene Blue molecules (grey) to the total wavefunction, as well as the population of the ground state (blue). Panel **e** shows the Mean Squared Displacement (*i.e.*, $\text{MSD}_{\text{exc}} = \langle z(t) - z(0)\rangle^2$) of the excitonic wavepacket depicted in Figure 3**e** of the main text (black line) and panel **b** (green line).

4.6 Simulations with constrained O-H bond in HBQ

To verify that a photo-chemical reaction is necessary for initiating polariton-assisted exciton transport, we keep the length of the O-H bond in the HBQ molecule fixed at 1 Å with a constraint.[14] As shown in Figure S9**a**, this constraint prevents excited-state proton transfer, and HBQ cannot relax into the global S_1 minimum that is resonant with the MeB molecules. As a consequence, there is no transfer of population into the MeB molecules (Figure S9**d**), and the exciton remains at the position of HBQ (Figure S9**b** and **e**). The minor decay of the HBQ population seen in Panel **c**, is due to population transfer into the high-energy cavity modes that are resonant with the HBQ when the O-H bond is constrained, but not with MeB.

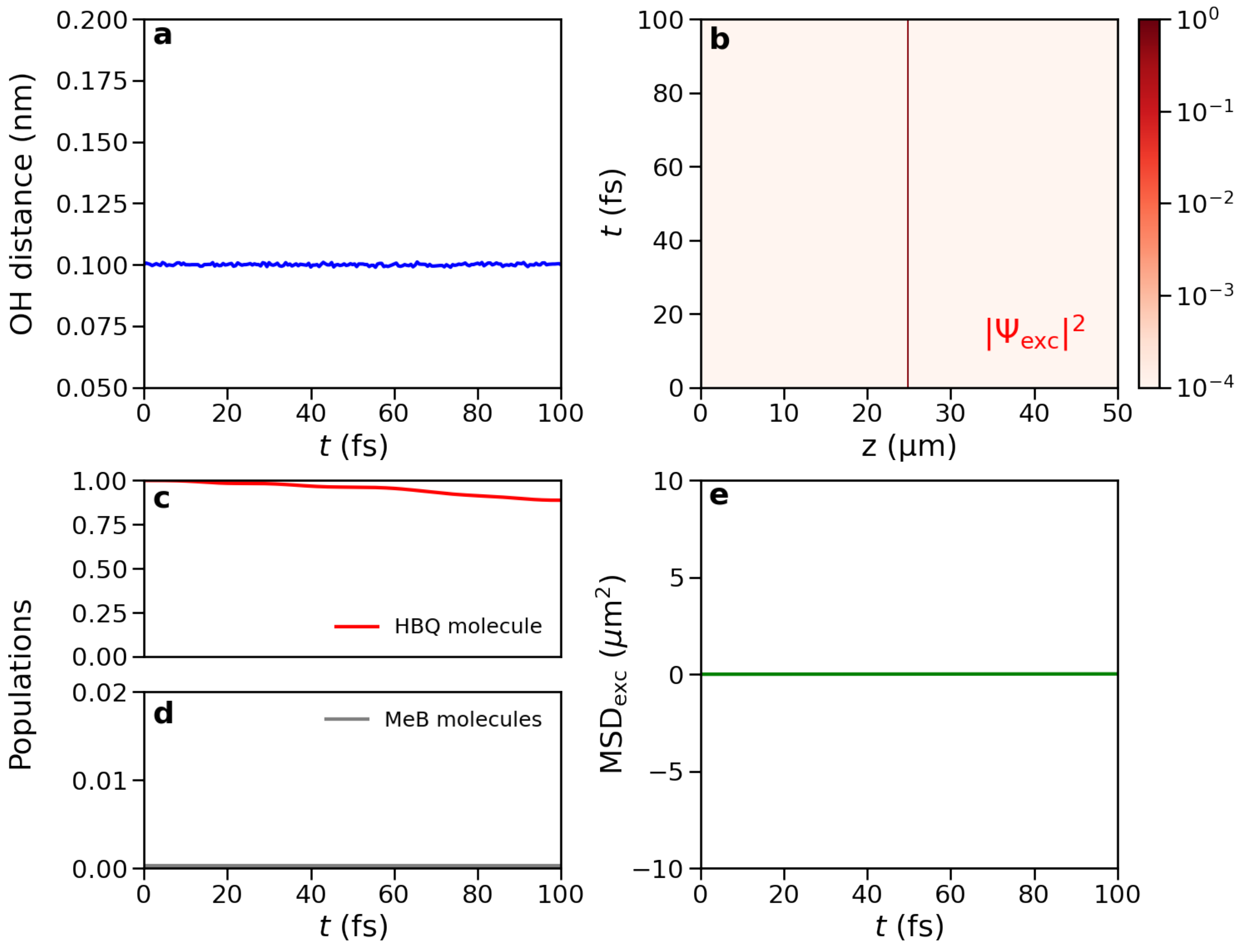

Figure S9: Polariton-mediated exciton transport when ESPIT in HBQ is blocked by a constraint on the O-H distance. Panel **a** shows the distance between the hydroxyl oxygen and proton, which we use as the reaction coordinate for the excited state proton transfer reaction (Figure S5), as a function of time after instantaneous excitation into the highest-energy eigenstate of the molecule-cavity system, which is dominated by the S_1 state of HBQ (*i.e.*, $|\beta_{\text{HBQ}}|^2 \approx 0.99$). Panel **b** shows the probability density of the excitonic part, $|\Psi_{\text{exc}}|^2$, of the total wavefunction as a function of the z-coordinate (horizontal axis) and time (vertical axis). Panels **c** and **d** show the contribution of the HBQ molecule (red) and Methylene Blue molecules (grey) to the total wavefunction, as well as the population of the ground state (blue). Panel **e** shows the Mean Squared Displacement (*i.e.*, $\text{MSD}_{\text{exc}} = \langle z(t) - z(0)\rangle^2$) of the excitonic wavepacket .

4.7 Effect of cavity decay

To investigate the effect of the Q-factor, we reduced the cavity decay rate from 66.7 ps^{-1} to 10 ps^{-1} and repeated the simulations. In addition, we also repeated the simulation for an ideal cavity with zero decay rate, and thus an infinite cavity mode lifetime. Comparing the results of these simulations, summarized in Figure 3 of the main text, and Figures S10 and S11 here, suggests that decreasing the cavity mode decay rate can significantly increase polariton-mediated exciton transport.

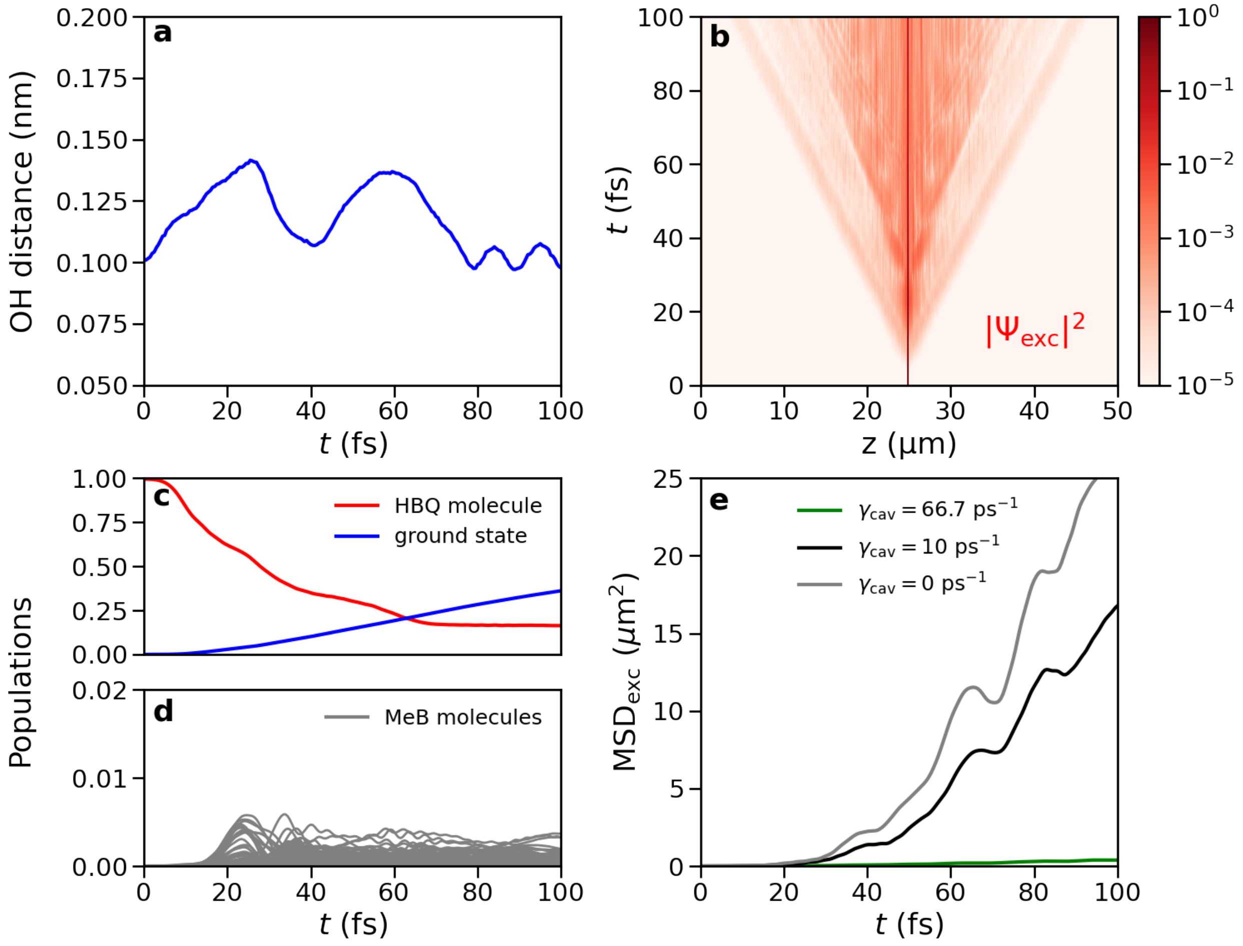

Figure S10: Polariton-mediated exciton transport in a cavity with the decay rate $\gamma_{\text{cav}} = 10\ \text{ps}^{-1}$. Panel **a** shows the distance between the hydroxyl oxygen and proton, which we use as the reaction coordinate for the excited state proton transfer reaction (Figure S5), as a function of time after instantaneous excitation into the highest-energy eigenstate of the molecule-cavity system, which is dominated by the S_1 state of HBQ (*i.e.*, $|\beta_{\text{HBQ}}|^2 \approx 0.99$). Panel **b** shows the probability density of the excitonic part, $|\Psi_{\text{exc}}|^2$, of the total wavefunction as a function of the z-coordinate (horizontal axis) and time (vertical axis). Panels **c** and **d** show the contribution of the HBQ molecule (red) and Methylene Blue molecules (grey) to the total wavefunction, as well as the population of the ground state (blue). Panel **e** shows the Mean Squared Displacement (*i.e.*, $\text{MSD}_{\text{exc}} = \langle z(t) - z(0) \rangle^2$) of the excitonic wavepacket in a cavity with the decay rate γ_{cav} of 66.7 ps^{-1} (green line), 10 ps^{-1} (black line) and 0 ps^{-1} (grey line).

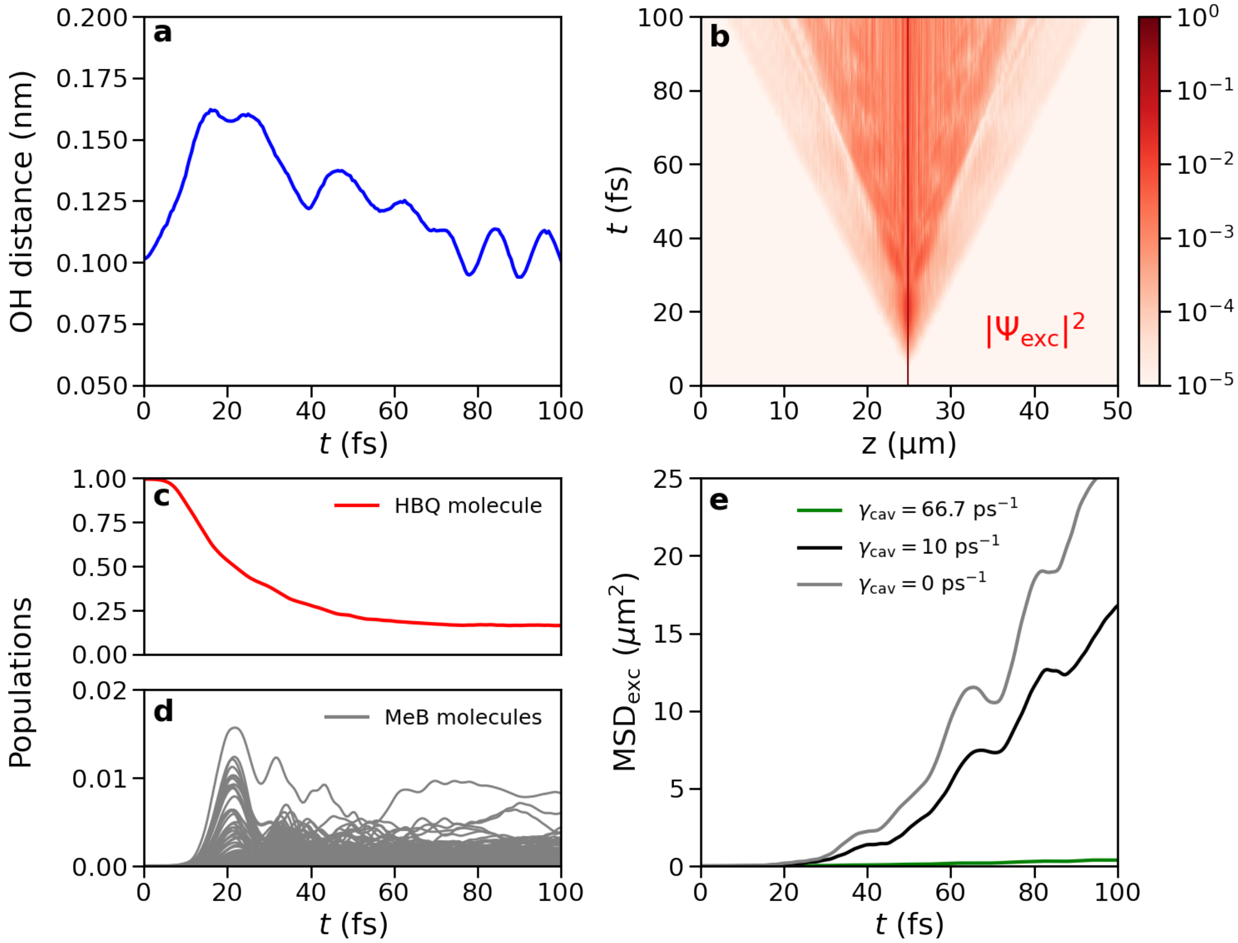

Figure S11: Polariton-mediated exciton transport in a perfect cavity, *i.e.*, $\gamma_{\text{cav}} = 0\ \text{ps}^{-1}$. Panel **a** shows the distance between the hydroxyl oxygen and proton, which we use as the reaction coordinate for the excited state proton transfer reaction (Figure S5), as a function of time after instantaneous excitation into the highest-energy eigenstate of the molecule-cavity system, which is dominated by the S_1 state of HBQ (*i.e.*, $|\beta_{\text{HBQ}}|^2 \approx 0.99$). Panel **b** shows the probability density of the excitonic part, $|\Psi_{\text{exc}}|^2$, of the total wavefunction as a function of the z-coordinate (horizontal axis) and time (vertical axis). Panels **c** and **d** show the contribution of the HBQ molecule (red) and Methylene Blue molecules (grey) to the total wavefunction, as well as the population of the ground state (blue). Panel **e** shows the Mean Squared Displacement (*i.e.*, $\text{MSD}_{\text{exc}} = \langle z(t) - z(0)\rangle^2$) of the excitonic wavepacket in a cavity with the decay rate γ_{cav} of 66.7 ps^{-1} (green line), 10 ps^{-1} (black line) and 0 ps^{-1} (grey line).

4.8 Blue-shifted polariton branches

To find out whether polarition propagation can be more efficiently initialized if the emission maximum of the HBQ overlaps with the lower polariton branch, rather than with the absorption maximum of MeB, we repeated the simulations with a constant offset of 250 meV added to the excitation energies of MeB and the fundamental cavity mode at $k_z = 0$ (Figure S12). Because this offset is constant, there are no changes in the gradients or transition dipole moments. The results of these simulations, shown in Figure S13, suggest no major effect on the propagation, which we attribute to the large line width of the HBQ emission spectrum.

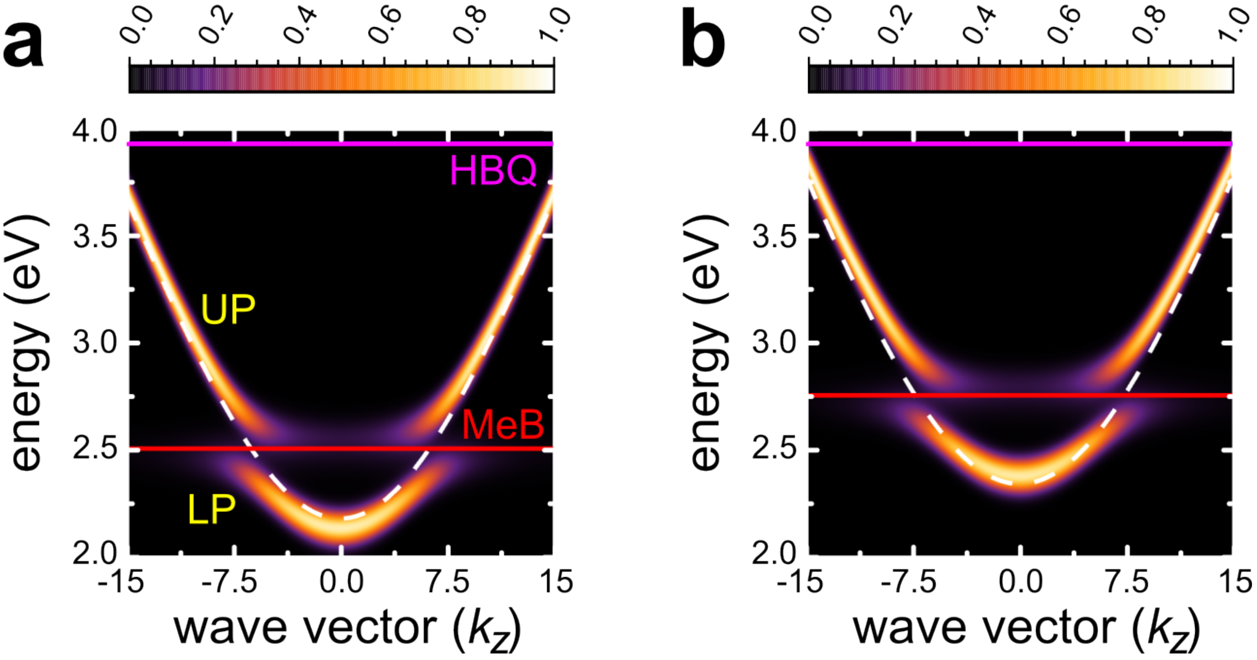

Figure S12: Panel **a**: normalised angle-resolved absorption spectrum of the molecule-cavity system. The cavity dispersion is plotted as a white dashed line, while the excitation maxima of the MeB molecules (∼2.5 eV) and HBQ are plotted as straight red and magenta lines, respectively. Panel **b**: normalised angle-resolved absorption spectrum of the molecule-cavity system, in which a constant offset of 250 meV was added to the excitation energy of the MeB molecules and to the fundamental cavity mode at $k_z = 0$. The cavity dispersion is plotted as a white dashed line, while the excitation maxima of the MeB molecules (∼2.75 eV with the offset) and HBQ are plotted as straight red and magenta lines, respectively.

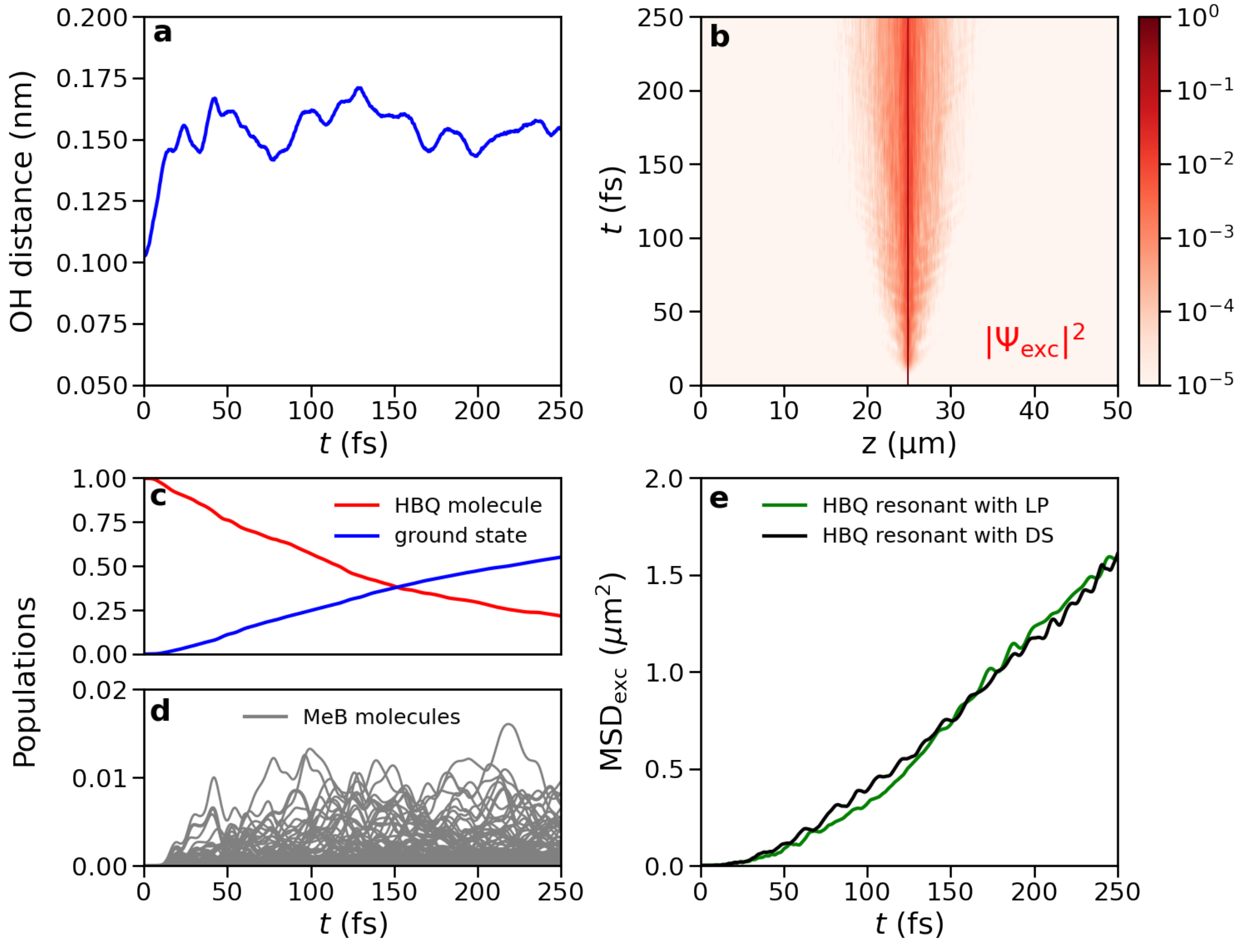

Figure S13: Polariton-mediated exciton transport when the emission maximum of HBQ overlaps with the lower polariton branch. Panel **a** shows the distance between the hydroxyl oxygen and proton, which we use as the reaction coordinate for the excited state proton transfer reaction (Figure S5), as a function of time after instantaneous excitation into the highest-energy eigenstate of the molecule-cavity system, which is dominated by the S_1 state of HBQ (*i.e.*, $|\beta_{\text{HBQ}}|^2 \approx 0.99$). Panel **b** shows the probability density of the excitonic part, $|\Psi_{\text{exc}}|^2$, of the total wavefunction as a function of the z-coordinate (horizontal axis) and time (vertical axis). Panels **c** and **d** show the contribution of the HBQ molecule (red) and Methylene Blue molecules (grey) to the total wavefunction, as well as the population of the ground state (blue). Panel **e** shows the Mean Squared Displacement (*i.e.*, $\text{MSD}_{\text{exc}} = \langle z(t) - z(0) \rangle^2$) of the excitonic wavepacket when the emission of HBQ is resonant with the lower polaritons (green line) or dark states (black line).

5 Animations

Animations of the excitonic wavepackets shown in Figure 3 of the main text (transport.mp4) and in Figure S8 (transport_delayed.mp4) are available for download in MPEG-4 format.

6 References

(1) Forn-Díaz, P.; Lamata, L.; Rico, E.; Kono, J.; Solano, E. Ultrastrong coupling regimes of light-matter interaction. *Rev. Mod. Phys.* **2019**, *91*, 025005.

(2) Jaynes, E. T.; Cummings, F. W. Comparison of quantum and semiclassical radiation theories with application to the beam maser. *Proc. IEEE* **1963**, *51*, 89–109.

(3) Tavis, M.; Cummings, F. W. Approximate solutions for an N-molecule radiation-field Hamiltonian. *Phys. Rev.* **1969**, *188*, 692–695.

(4) Michetti, P.; Rocca, G. C. L. Polariton states in disordered organic microcavities. *Phys. Rev. B.* **2005**, *71*, 115320.

(5) Tichauer, R. H.; Feist, J.; Groenhof, G. Multi-scale Dynamics Simulations of Molecular Polaritons: the Effect of Multiple Cavity Modes on Polariton Relaxation. *J. Chem. Phys.* **2021**, *154*, 104112.

(6) Warshel, A.; Levitt, M. Theoretical studies of enzymatic reactions: Dielectric, electrostatic and steric stabilization of carbonium ion in the reaction of lysozyme. *J. Mol. Biol.* **1976**, *103*, 227–249.

(7) Boggio-Pasqua, M.; Burmeister, C. F.; Robb, M. A.; Groenhof, G. Photochemical reactions in biological systems: probing the effect of the environment by means of hybrid quantum chemistry/molecular mechanics simulations. *Phys. Chem. Chem. Phys.* **2012**, *14*, 7912–7928.

(8) Ehrenfest, P. Bemerkung über die angenäherte Gültigkeit der klassischen Mechanik innerhalb der Quantenmechanik. *Z. Phys.* **1927**, *45*, 445–457.

(9) Felicetti, S.; Fregoni, J.; Schnappinger, T.; Reiter, S.; de Vivie-Riedle, R.; Feist, J. Photoprotecting Uracil by Coupling with Lossy Nanocavities. *J. Chem. Phys. Lett.* **2020**, *11*, 8810–8818.

(10) Ulusoy, I. S.; Vendrell, O. Dynamics and spectroscopy of molecular ensembles in a lossy microcavity. *J. Chem. Phys.* **2020**, *153*, 044108.

(11) Antoniou, P.; Suchanek, F.; Varner, J. F.; Foley IV, J. J. Role of Cavity Losses on Nonadiabatic Couplings and Dynamics in Polaritonic Chemistry. *J. Phys. Chem. Lett.* **2020**, *11*, 9063–9069.

(12) Hu, D.; Mandal, A.; Weight, B. M.; Huo, P. Quasi-diabatic propagation scheme for simulating polariton chemistry. *J. Chem. Phys.* **2022**, *157*, 194109.

(13) Horta, B. A. C.; Merz, P. T.; Fuchs, P. F. J.; Dolenc, J.; Riniker, S.; Hünenberger, P. H. A GROMOS-Compatible Force Field for Small Organic Molecules in the Condensed Phase: The 2016H66 Parameter Set. *J. Chem. Theory. Comput.* **2016**, *12*, 3825–3850.

(14) Hess, B.; Bekker, H.; Berendsen, H. J. C.; Fraaije, J. G. E. M. LINCS: A linear constraint solver for molecular simulations. *J. Comput. Chem.* **1997**, *18*, 1463–1472.

(15) Berendsen, H.; Postma, J.; van Gunsteren, W.; la, A. D.; Haak, J. Molecular dynamics with coupling to an external bath. *J. Chem. Phys.* **1984**, *81*, 3684–3690.

(16) Becke, A. D. A new mixing of Hartree-Fock and local density-functional theories. *J. Chem. Phys.* **1993**, *98*, 1372.

(17) Lee, C. T.; Yang, W. T.; Parr, R. G. Development of the Colle-Salvet correlation-energy formula into a functional of the electron density. *Phys. Rev. B* **1988**, *37*, 785–789.

(18) Yanai, T.; Tew, D. P.; Handy, N. C. A new hybrid exchange-correlation functional using the Coulomb-attenuating method (CAM-B3LYP). *Chem. Phys. Lett.* **2004**, *393*, 51–57.

(19) Dunning, T. H. Basis Functions for Use in Molecular Calculations. I. Contractions of (9s5p) Atomic Basis Sets for the First-Row Atoms. *J. Chem. Phys.* **1970**, *53*, 2823–2833.

(20) Runge, E.; Gross, E. K. U. Density-Functional Theory for Time-Dependent Systems. *Phys. Rev. Lett* **1984**, *52*, 997–1000.

(21) Hirata, S.; Head-Gordon, M. Time-dependent density functional theory within the Tamm–Dancoff approximation. *Chem. Phys. Lett.* **1999**, *314*, 291–299.

(22) Hess, B.; Kutzner, C.; van der Spoel, D.; Lindahl, E. GROMACS 4: Algorithms for Highly Efficient, Load-Balanced, and Scalable Molecular Simulation. *J. Chem. Theory Comput.* **2008**, *4*, 435–447.

(23) Ufimtsev, I. S.; Martínez, T. J. Quantum Chemistry on Graphical Processing Units. 3. Analytical Energy Gradients and First Principles Molecular Dynamics. *J. Chem. Theory Comput.* **2009**, *5*, 2619–2628.

(24) Titov, A. V.; Ufimtsev, I. S.; Luehr, N.; Martínez, T. J. Generating Efficient Quantum Chemistry Codes for Novel Architectures. *J. Chem. Theory Comput.* **2013**, *9*, 213–221.

(25) Jorgensen, W. L.; Chandrasekhar, J.; Madura, J. D.; Impey, R. W.; Klein, M. L. Comparison of simple potential functions for simulatin liquid water. *J. Chem. Phys.* **1983**, *79*, 926–935.

(26) Duan, Y.; Wu, C.; Chowdhury, S.; Lee, M. C.; Xiong, G. M.; Zhang, W.; Yang, R.; Cieplak, P.; Luo, R.; Lee, T.; Caldwell, J.; Wang, J. M.; Kollman, P. A point-charge force field for molecular mechanics simulations of proteins based on condensed-phase quantum mechanical calculations. *J. Comput. Chem.* **2003**, *24*, 1999–2012.

(27) Bayly, C. I.; Cieplak, P.; Cornell, W. D.; Kollman, P. A. A well-behaved electrostatic potential bsed method using charge restraints for deriving atomic charges - the RESP model. *J. Phys. Chem.* **1993**, *97*, 10269–10280.

(28) Tomasi, J.; Mennucci, B.; Cammi, R. Quantum Mechanical Continuum Solvation Models. *Chem. Rev.* **2005**, *105*, 2999–3094.

(29) Essmann, U.; Perera, L.; Berkowitz, M. L.; Darden, T.; Lee, H.; Pedersen, L. G. A smooth particle mesh Ewald potential. *J. Chem. Phys* **1995**, *103*, 8577–8592.

(30) Bussi, G.; Donadio, D.; Parrinello, M. Canonical sampling through velocity rescaling. *J. Chem. Phys.* **2007**, *126*, 014101.

(31) Miyamoto, S.; Kollman, P. A. SETTLE: An analytical version of the SHAKE and RATTLE algorithms for rigid water molecules. *J. Comp. Chem.* **1992**, *18*, 1463–1472.

(32) Becke, A. D. Density-functional thermochemistry. V. Systematic optimization of exchange-correlation functionals. *J. Chem. Phys.* **1997**, *107*, 8554–8560.

(33) Modi, V.; Donnini, S.; Groenhof, G.; Morozov, D. Protonation of the Biliverdin IXα Chromophore in the Red and Far- Red Photoactive States of a Bacteriophytochrome. *J. Phys. Chem. B* **2019**, *123*, 2325–2334.

(34) Agranovich, V. M.; Gartstein, Y. N. Nature and Dynamics of Low-Energy Exciton Polaritons in Semiconductor Microcavities. *Phys. Rev. B* **2007**, *75*, 075302.

(35) Lidzey, D. G.; Bradley, D. C.; Armitage, A.; Walker, S.; Skolnick, M. S. Photon-Mediated Hybridization of Frenkel Excitons in Organic Semiconductor Microcavities. *Science* **2000**, *288*, 1620–1623.

(36) Dutta, A.; Tiainen, V.; Duarte, L.; Markesevic, N.; Morozov, D.; Qureshi, H. A.; Groenhof, G.; Toppari, J. J. Ultra-fast photochemistry in the strong light-matter coupling regime. *Chemrxiv* **2023**,

(37) Staroverov, V. N.; Scuseria, G. E.; Tao, J.; Perdew, J. P. Comparative assessment of a new nonempirical density functional: Molecules and hydrogen-bonded complexes. *J. Chem. Phys.* **2003**, *119*, 12129–12137.

(38) Picconi, D. Nonadiabatic quantum dynamics of the coherent excited state intramolecular proton transfer of 10-hydroxybenzo [h] quinoline. *Photochem. Photobiol. Sci.* **2021**, *20*, 1455–1473.
